# Supplementary material for: Probabilistic forecasting of replication studies
Source: PLoS One. 2020 Apr 22;15(4):e0231416. doi: 10.1371/journal.pone.0231416 (PMC7176099; doi:10.1371/journal.pone.0231416)
Supplement: S1 Appendix — Description and code for preprocessing of data. (PDF) [file pone.0231416.s001.pdf]

Probabilistic forecasting of replication studies
Supplement A: Data preprocessing

Samuel Pawel, Leonhard Held

October 28, 2019

1 Effect size scale

In the analysis of data from replication projects it has become common practice to transform effect sizes to the correlation coefficient scale r (Open Science Collaboration, 2015; Camerer et al., 2016, 2018; Cova et al., 2018). For an introduction to the conversion between effect size scales, see section 12.5 in Cooper et al. (2009). An advantage of correlation coefficients is that they are bounded to the interval between minus one and one and are thus easy to compare and interpret. Moreover, by applying the variance stabilizing transformation, also known as Fisher z-transformation, $\hat{\theta} = \tanh^{-1}(r)$, the transformed correlation coefficients become asymptotically normally distributed with their variance only being a function of the study sample size n, *i. e.* $\text{Var}(\hat{\theta}) = 1/(n-3)$ (Fisher, 1921). The Fisher z-transformation is shown in Figure 1. Throughout the paper modelling and prediction is carried out on the Fisher z scale, but the results are often be backtransformed to the correlation scale by applying the inverse Fisher z-transformation, $r = \tanh(\hat{\theta})$, for better comparability and interpretability.

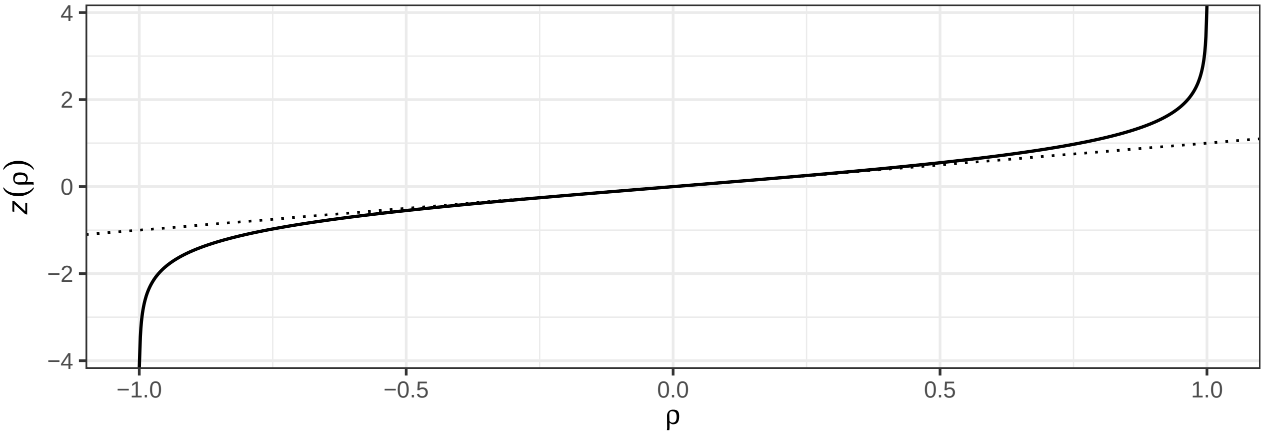

Figure 1: Fisher z-transformation.

Open Science Collaboration (2015) used the approach of computing *correlation per degree of freedom* based on the test statistics of the original effect estimates (see page 74 in the supplementary material of Open Science Collaboration, 2015). This is possible for z, χ^2, t, and F test statistics and can be done using the following formulas

$$\rho(t) = \frac{\sqrt{t^2/\text{df}_2}}{(t^2/\text{df}_2) + 1}$$
$$\rho(F) = \frac{\sqrt{F(\text{df}_1/\text{df}_2)}}{\{F(\text{df}_1/\text{df}_2) + 1\}\sqrt{1/\text{df}_1}}$$
$$\rho(\chi^2) = \sqrt{\chi^2/n}$$
$$\rho(z) = \tanh\left(z\sqrt{\frac{1}{n-3}}\right).$$

The approach has become the standard for further replication projects (Camerer et al., 2016, 2018; Cova et al., 2018).

2 Data preprocessing

2.1 Reproducibility Project: Psychology

All files were downloaded from `https://github.com/CenterForOpenScience/rpp/archive/master.zip`. The `masterscript.R` was run and the data then taken from the generated `MASTER` object. The standard errors of the Fisher z-transformed correlation coefficients were obtained by binding the `final$sei.o` and `final$sei.r` vectors with the remaining data. According to the supplementary material, the p-values from the studies with ID's 7, 15, 47, 94, 120, and 140 were one-sided and were therefore multiplied by two to obtain two-sided p-values. Only the "meta-analytic subset" was used, which consists of 73 studies where the standard error of the Fisher z-transformed effect estimates can be computed.

```
# ==============================================================================
# Reproducibility project Psychology (rpp)
# ==============================================================================
library(dplyr)
library(readr)
url_master <- "https://github.com/CenterForOpenScience/rpp/archive/master.zip"
download.file(url = url_master, destfile = "rpp_git_repo.zip")
unzip("rpp_git_repo.zip" )

# ATTENTION: if running on linux: comment out the windows command
# "choose.dir" at beginning of masterscript.R !
setwd("rpp-master/")
source("masterscript.R")
setwd("../")

MASTER_cleaned <- MASTER %>%
  mutate(FZ_OS = atanh(T_r..O.),
         FZ_RS = atanh(T_r..R.),
         Actual.Power..O. = as.double(as.character(Actual.Power..O.)),
         Power..R. = as.double(Power..R.)) %>%
  select(ID,
         Authors..O.,
         Journal..O.,
         Discipline..O.,
         T_Test.Statistic..O.,
         T_df1..O.,
         T_df2..O.,
         T_Test.value..O.,
         Type.of.analysis..O.,
         Effect.size..O.,
         Actual.Power..O.,
         T_N..O.,
         T_r..O.,
         T_pval_USE..O.,
         FZ_OS,
         T_Test.Statistic..R.,
         T_df1..R.,
         T_df2..R.,
         T_Test.value..R.,
         Type.of.analysis..R.,
         Power..R.,
         Effect.Size..R.,
         T_N..R.,
         T_r..R.,
         T_pval_USE..R.,
         FZ_RS,
         Meta.analytic.estimate..Fz.) %>%
  rename(Study_ID = ID,
         Authors_OS = Authors..O.,
```

```
         Journal_OS = Journal..O.,
         Discipline = Discipline..O.,
         Type_Test_Statistic_OS = T_Test.Statistic..O.,
         DF1_OS = T_df1..O.,
         DF2_OS = T_df2..O.,
         Test_Statistic_OS = T_Test.value..O.,
         N_OS = T_N..O.,
         r_OS = T_r..O.,
         pval_OS = T_pval_USE..O.,
         Analysis_Type_OS = Type.of.analysis..O.,
         Effect_Size_OS = Effect.size..O.,
         Power_OS = Actual.Power..O.,
         Type_Test_Statistic_RS = T_Test.Statistic..R.,
         DF1_RS = T_df1..R.,
         DF2_RS = T_df2..R.,
         Test_Statistic_RS = T_Test.value..R.,
         N_RS = T_N..R.,
         r_RS = T_r..R.,
         pval_RS = T_pval_USE..R.,
         Analysis_Type_RS = Type.of.analysis..R.,
         Effect_Size_RS = Effect.Size..R.,
         Power_RS = Power..R.,
         FZ_meta = Meta.analytic.estimate..Fz.)

MASTER_cleaned$FZ_se_OS[!is.na(MASTER_cleaned$FZ_meta)] <- final$sei.o
MASTER_cleaned$FZ_se_RS[!is.na(MASTER_cleaned$FZ_meta)] <- final$sei.r
MASTER_cleaned <- MASTER_cleaned %>%
  # these were one-sided p-values according supplementary
  mutate(pval_OS = ifelse(Study_ID %in% c(7, 15, 47, 94, 120, 140),  pval_OS*2, pval_OS),
         pval_OS = ifelse(pval_OS > 1, 1, pval_OS),
         pval_RS = ifelse(Study_ID %in% c(7, 15, 47, 94, 120, 140), pval_RS*2, pval_RS),
         pval_RS = ifelse(pval_RS > 1, 1, pval_RS),
         pvalFZ_OS = 2*pnorm(FZ_OS/FZ_se_OS, lower.tail = FALSE),
         pvalFZ_OS = ifelse(pvalFZ_OS > 1, 1, pvalFZ_OS),
         pvalFZ_RS = 2*pnorm(FZ_RS/FZ_se_RS, lower.tail = FALSE),
         pvalFZ_RS = ifelse(pvalFZ_RS > 1, 1, pvalFZ_RS))
write_csv(MASTER_cleaned, path = "RPP.csv")
```

2.2 Experimental Economics Replication Project

All files were downloaded from `https://osf.io/pnwuz/`. However, to "generate" the data from the file `create_studydetails.do`, the commercial software `STATA` is required. Since the data set is very small, the required data were manually extracted from the code in the file `create_studydetails.do`. To compute the standard errors of the Fisher z-transformed effect estimates, the sample sizes reported in the `effectdata.py` file rather than the ones reported in the `create_studydetails.do` were taken. The former correspond to the effective sample sizes while the latter in some cases corresponds to the number of measurements, which lead to different prediction intervals than the ones reported in the publication (however, in all tables Camerer et al. (2016) report the larger "number of measurements" sample size). The data regarding the prediction market and survey beliefs were also manually extracted from table S3 in the supplementary material, which was downloaded from `http://science.sciencemag.org/content/suppl/2016/03/02/science.aaf0918.DC1`.

```
# ==============================================================================
# Experimental economics replication project (eerp)
# ==============================================================================
# - All files downloaded from https://osf.io/pnwuz/
# - Unfortunately this data had to be manually recorded from the file
#   "create_studydetails.do" since I do not own the commercial software stata
#   which is required to run the .do file and generate .dat file (economists ..)
# - Similarly, effective sample size taken from  "effectstandardization.py" file
# - Prediction market infos taken from table S3 in Supplementary of Article
```

```r
#   http://science.sciencemag.org/content/sci/suppl/2016/03/02/
#   science.aaf0918.DC1/aaf0918-Camerer-SM.pdf

library(dplyr)
library(readr)
Study <- c("Abeler et al. (AER 2011)",
           "Ambrus and Greiner (AER 2012)",
           "Bartling et al. (AER 2012)",
           "Charness and Dufwenberg (AER 2011)",
           "Chen and Chen (AER 2011)",
           "de Clippel et al. (AER 2014)",
           "Duffy and Puzzello (AER 2014)",
           "Dulleck et al. (AER 2011)",
           "Fehr et al. (AER 2013)",
           "Friedman and Oprea (AER 2012)",
           "Fudenberg et al. (AER 2012)",
           "Huck et al. (AER 2011)",
           "Ifcher and Zarghamee (AER 2011)",
           "Kessler and Roth (AER 2012)",
           "Kirchler et al (AER 2012)",
           "Kogan et al. (AER 2011)",
           "Kuziemko et al. (QJE 2014)",
           "Ericson and Fuster (QJE 2011)")
Market_Belief <- c(0.696, 0.692, 0.805, 0.695, 0.778, 0.759, 0.806, 0.738, 0.629,
                   0.833, 0.933, 0.920, 0.588, 0.937, 0.712, 0.802, 0.632, 0.622)
Survey_Belief_premarket <- c(0.696, 0.542, 0.807, 0.715, 0.682, 0.730, 0.685,
                             0.807, 0.674, 0.863, 0.790, 0.749, 0.542, 0.837,
                             0.704, 0.748, 0.568, 0.658)
Survey_Belief_postmarket <- c(0.697, 0.620, 0.733, 0.708, 0.692, 0.716, 0.694,
                              0.744, 0.666, 0.817, 0.770, 0.730, 0.566, 0.825,
                              0.728, 0.752, 0.582, 0.650)
pval_OS <- c(0.046, 0.057, 0.007, 0.01, 0.033, 0.001, 0.01, 0.0001, 0.011,
             4*10^(-11), 0.001, 0.0039, 0.031, 1.631*10^(-18), 0.0163, 0.000026,
             0.07, 0.03)
# Attention, these are session numbers, not effective sample size!
# N_OS <- c(120, 117, 216, 162, 72, 158, 54, 168, 60, 78, 124,120, 58, 288, 120,
#           126, 42, 112)
# These are effective sample size:
N_OS <- c(120, 39, 12, 43, 6, 790, 9, 21, 30, 78, 124, 12, 58, 288, 12, 160,
          42, 104)
r_OS <- c(0.182821975588, 0.310518647505, 0.719849875686, 0.383943377571,
          0.842508557739, 0.117768981826, 0.761510174904, 0.722509234548,
          0.453281944406, 0.642590125822, 0.303741608956, 0.832065702534,
          0.282103220856, 0.486223364603, 0.664409308738, 0.323896842962,
          0.282261740933, 0.212921823047)
pval_RS <- c(0.16, 0.012, 0.001, 0.003, 0.571, 4*10^(-10), 0.674, 0.0008,
             0.026, 0.004276, 0.0001506473, 0.1415, 0.933, 0.016, 0.0095,
             0.001, 0.154, 0.0546)
# Attention, these are session numbers, not effective sample size!
# N_RS <- c(318, 357, 360, 264, 168, 156, 96, 128, 102, 40, 128, 160, 131, 48,
#           220, 90, 144, 262)
# These are effective sample size:
N_RS <- c(318, 119, 20, 65, 14, 780, 16, 16, 51, 40, 128, 16, 131, 48, 22,
          112, 144, 248)
r_RS <- c(0.0790703532018, 0.229536356959, 0.657411288278, 0.363002684809,
          0.170189166838, 0.266538269195, -0.11596300548, 0.731605727911,
          0.311199311719, 0.437953607707, 0.326573539422, 0.367593525514,
          -0.00701629144787, 0.34463841128, 0.533556821497, 0.304223231247,
          -0.119848901099, 0.122871403263)
Power_RS <- c(0.9, 0.91, 0.94, 0.9, 0.9, 0.9, 0.93, 0.92, 0.91, 0.99, 0.92,
```

```r
              0.91, 0.9, 0.95, 0.9, 0.94, 0.92, 0.91)

tibble(Study, r_OS, N_OS, pval_OS, r_RS, N_RS, pval_RS, Power_RS,
       Market_Belief, Survey_Belief_premarket, Survey_Belief_postmarket) %>%
  mutate(FZ_OS = atanh(r_OS),
         FZ_se_OS = 1/sqrt(N_OS - 3),
         pvalFZ_OS = 2*pnorm(FZ_OS/FZ_se_OS, lower.tail = FALSE),
         pvalFZ_OS = ifelse(pvalFZ_OS > 1, 1, pvalFZ_OS),
         FZ_RS = atanh(r_RS),
         FZ_se_RS = 1/sqrt(N_RS - 3),
         pvalFZ_RS = 2*pnorm(FZ_RS/FZ_se_RS, lower.tail = FALSE),
         pvalFZ_OS = ifelse(pvalFZ_RS > 1, 1, pvalFZ_RS)) %>%
  write_csv(path = "EERP.csv")
```

2.3 Social Sciences Replication Project

The data were taken from the `D3 - ReplicationResults.csv` file, which was downloaded from `https://osf.io/abu7k`. For replications which underwent only the first stage, the data from the first stage were taken as the data for the replication study. For the replications which reached the second stage, the pooled data from both stages were taken as the data for the replication study. Additionally, the data regarding survey and prediction market beliefs were extracted from the `D6 - MeanPeerBeliefs.csv` file, which was downloaded from `https://osf.io/vr6p8/`.

```r
# ============================================================================
# Social sciences replication project (ssrp)
# ============================================================================
library(dplyr)
library(readr)
names <- c("Ackerman et al. (2010), Science",
           "Aviezer et al. (2012), Science",
           "Balafoutas and Sutter (2012), Science",
           "Derex et al. (2013), Nature",
           "Duncan et al. (2012), Science",
           "Gervais and Norenzayan (2012), Science",
           "Gneezy et al. (2014), Science",
           "Hauser et al. (2014), Nature",
           "Janssen et al. (2010), Science",
           "Karpicke and Blunt (2011), Science",
           "Kidd and Castano (2013), Science",
           "Kovacs et al. (2010), Science",
           "Lee and Schwarz (2010), Science",
           "Morewedge et al. (2010), Science",
           "Nishi et al. (2015), Nature",
           "Pyc and Rawson (2010), Science",
           "Ramirez and Beilock (2011), Science",
           "Rand et al. (2012), Nature",
           "Shah et al. (2012), Science",
           "Sparrow et al. (2011), Science",
           "Wilson et al. (2014), Science")
download.file(url = "https://osf.io/abu7k/download",
              destfile = "SSRP_Data_Processed.csv")
download.file(url = "https://osf.io/vr6p8/download",
              destfile = "SSRP_Data_Peer_Beliefs_Processed.csv")
ssrp <- read_csv("SSRP_Data_Processed.csv")
ssrp_pmarket <- read_csv("SSRP_Data_Peer_Beliefs_Processed.csv")
prediction_markets <- ssrp_pmarket %>%
  select(m3_p, m3_b) %>%
  rename(Market_Belief = m3_p,
         Survey_Belief = m3_b) %>%
  filter(!is.na(Market_Belief))
```

```
ssrp %>%
  mutate(Name_OS = names,
         FZ_OS = atanh(r_os),
         FZ_se_OS = 1/sqrt(n_os - 3),
         FZ_RS1 = atanh(r_rs1),
         FZ_se_RS1 = 1/sqrt(n_rs1 - 3),
         FZ_RS2 = atanh(r_rs2),
         FZ_se_RS2 = 1/sqrt(n_rs2 - 3)) %>%
  select(study,
         Name_OS,
         sref,
         type_os,
         stat_os,
         n_os,
         in_os,
         r_os,
         r95l_os,
         r95u_os,
         p_os,
         FZ_OS,
         FZ_se_OS,
         type_rs1,
         stat_rs1,
         n_rs1,
         in_rs1,
         r_rs1,
         r95l_rs1,
         r95u_rs1,
         p_rs1,
         pow_rs1,
         FZ_RS1,
         FZ_se_RS1,
         type_rs2,
         stat_rs2,
         n_rs2,
         in_rs2,
         r_rs2,
         r95l_rs2,
         r95u_rs2,
         p_rs2,
         pow_rs2,
         FZ_RS2,
         FZ_se_RS2) %>%
  rename(Study = study,
         Sref = sref,
         Type_OS = type_os,
         Stat_OS = type_os,
         N_OS = n_os,
         In_OS = in_os,
         r_OS = r_os,
         r95l_OS = r95l_os,
         r95u_OS = r95u_os,
         pval_OS = p_os,
         Type_RS1 = type_rs1,
         Stat_RS1 = stat_rs1,
         N_RS1 = n_rs1,
         In_RS1 = in_rs1,
         r_RS1 = r_rs1,
         r95l_RS1 = r95l_rs1,
         r95u_RS1 = r95u_rs1,
```

```
        pval_RS1 = p_rs1,
        Power_RS1 = pow_rs1,
        Type_RS2 = type_rs2,
        Stat_RS2 = stat_rs2,
        N_RS2 = n_rs2,
        In_RS2 = in_rs2,
        r_RS2 = r_rs2,
        r95l_RS2 = r95l_rs2,
        r95u_RS2 = r95u_rs2,
        pval_RS2 = p_rs2,
        Power_RS2 = pow_rs2) %>%
  mutate(r_RS = ifelse(!is.na(r_RS2), r_RS2, r_RS1),
         FZ_RS = ifelse(!is.na(FZ_RS2), FZ_RS2, FZ_RS1),
         FZ_se_RS = ifelse(!is.na(FZ_se_RS2), FZ_se_RS2, FZ_se_RS1),
         pval_RS = ifelse(!is.na(FZ_se_RS2), pval_RS2, pval_RS1),
         N_RS = ifelse(!is.na(FZ_se_RS2), N_RS2, N_RS1)) %>%
  bind_cols(., prediction_markets) %>%
  write_csv(path = "SSRP.csv")
```

2.4 Experimental Philosophy Replicability Project

All data were taken from the `XPhiReplicability_CompleteData.csv` file, which was downloaded from `https://osf.io/4ewkh`. However, only a subset of 31 of these replications could be used, since only for these data, effect estimates on correlation scale and effective sample size for original and replication were available simultaneously. Because p-values were most of the time reported as inequalities, they were recalculated using a normal approximation on the Fisher z scale.

```
# ==============================================================================
# Experimental philosophy replicability project (rpphi)
# ==============================================================================
library(dplyr)
library(readr)
download.file(url = "https://osf.io/4ewkh/download",
              destfile = "XPhiReplicability_CompleteData.csv")
rpphi <- read_csv("XPhiReplicability_CompleteData.csv")
rpphi %>%
  mutate(FZ_OS = atanh(OriginalRES),
         FZ_RS = atanh(ReplicationRES),
         FZ_se_OS = 1/sqrt(OriginalN_Effect - 3),
         FZ_se_RS = 1/sqrt(ReplicationN_Effect - 3),
         pval_RS = 2*pnorm(abs(FZ_RS/FZ_se_RS), lower.tail = FALSE),
         pval_RS = ifelse(pval_RS > 1, 1, pval_RS),
         pval_OS = 2*pnorm(abs(FZ_OS/FZ_se_OS), lower.tail = FALSE),
         pval_OS = ifelse(pval_OS > 1, 1, pval_OS)) %>%
  select(PAPER_ID,
         OriginalN_Effect,
         OriginalTEST,
         OriginalEFFECTSIZE,
         OriginalANALYSIS,
         OriginalRES,
         OriginalPOWER,
         OriginalR95CI,
         FZ_OS,
         FZ_se_OS,
         pval_OS,
         ReplicationN_Effect,
         ReplicationTEST,
         ReplicationANALYSIS,
         ReplicationEFFECTSIZE,
         ReplicationRES,
```

```
        ReplicationR95CI,
        FZ_RS,
        FZ_se_RS,
        pval_RS,
        ReplicationSUCCESS,
        OSF) %>%
  rename(Study = PAPER_ID,
        Type_Test_OS = OriginalTEST,
        Test_Statistic_OS = OriginalANALYSIS,
        N_OS = OriginalN_Effect,
        r_OS = OriginalRES,
        r_CI_OS = OriginalR95CI,
        Effect_Size_OS = OriginalEFFECTSIZE,
        Power_OS = OriginalPOWER,
        Type_Test_RS = ReplicationTEST,
        Test_Statistic_RS = ReplicationANALYSIS,
        N_RS = ReplicationN_Effect,
        r_RS = ReplicationRES,
        r_CI_RS = ReplicationR95CI,
        Effect_Size_RS = ReplicationEFFECTSIZE,
        Replication_Success = ReplicationSUCCESS) %>%
 write_csv(path = "RPPHI.csv")
```

```
# ============================================================================
# Combining all data sets
# ============================================================================
library(dplyr)
library(readr)
rpp <- read_csv("RPP/RPP.csv")
rpphi <- read_csv("RPPHI/RPPHI.csv")
ssrp <- read_csv("SSRP/SSRP.csv")
eerp <- read_csv("EERP/EERP.csv")

# Subsets of data where effect sizes transformed to correlations available
rpp_correlations_subset <- rpp %>%
  filter(!is.na(r_OS) & !is.na(r_RS)) %>%
  mutate(Project = "Psychology",
        Study = Authors_OS,
        Survey_Belief_Premarket = NA,
        Survey_Belief_Postmarket = NA,
        Market_Belief = NA) %>%
  select(Study, r_OS, r_RS, FZ_OS, FZ_RS, FZ_se_OS, FZ_se_RS, pval_RS,
        N_OS, N_RS, pval_OS, Project, Survey_Belief_Premarket,
        Survey_Belief_Postmarket, Market_Belief)

eerp_correlations_subset <- eerp %>%
  mutate(Project = "Experimental Economics",
        Stuy = Study,
        Survey_Belief_Premarket = eerp$Survey_Belief_premarket,
        Survey_Belief_Postmarket = eerp$Survey_Belief_postmarket,
        Market_Belief = eerp$Market_Belief) %>%
  select(Study, r_OS, r_RS, FZ_OS, FZ_RS, FZ_se_OS, FZ_se_RS, pval_RS,
        N_OS, N_RS, pval_OS, Project, Survey_Belief_Premarket,
        Survey_Belief_Postmarket, Market_Belief)

ssrp_correlations_subset <- ssrp %>%
  mutate(Project = "Social Sciences",
        Study = Name_OS,
        Survey_Belief_Premarket = ssrp$Survey_Belief,
        Survey_Belief_Postmarket = NA,
```

```r
         Market_Belief = ssrp$Market_Belief) %>%
  select(Study, r_OS, r_RS, FZ_OS, FZ_RS, FZ_se_OS, FZ_se_RS, pval_RS,
         N_OS, N_RS, pval_OS, Project, Survey_Belief_Premarket,
         Survey_Belief_Postmarket, Market_Belief)

rpphi_correlations_subset <- rpphi %>%
  filter(!is.na(r_OS) & !is.na(r_RS) & !is.na(pval_RS)) %>%
  mutate(Project = "Experimental Philosophy",
         Study = Study,
         Survey_Belief_Premarket = NA,
         Survey_Belief_Postmarket = NA,
         Market_Belief = NA) %>%
  select(Study, r_OS, r_RS, FZ_OS, FZ_RS, FZ_se_OS, FZ_se_RS, pval_RS,
         N_OS, N_RS, pval_OS, Project, Survey_Belief_Premarket,
         Survey_Belief_Postmarket, Market_Belief)

data_correlations_subset <- rbind(rpp_correlations_subset,
                                  eerp_correlations_subset,
                                  ssrp_correlations_subset,
                                  rpphi_correlations_subset) %>%
  mutate(pval_RS_significant = factor(pval_RS < 0.05,
                                      labels = c("Not Significant",
                                                 "Significant")))

write_csv(data_correlations_subset,
          path = "Data_Final/replications_correlation_subset.csv")

# Subset of data where standard error of Fisher z-transformed correlations available (Meta analytic sub
data_ma_subset <- data_correlations_subset %>%
  filter(!is.na(FZ_se_OS) & !is.na(FZ_se_RS))

write_csv(data_ma_subset, path = "Data_Final/replications_ma_subset.csv")
```

References

Camerer, C., Dreber, A., Holzmeister, F., Ho, T., Huber, J., Johannesson, M., Kirchler, M., Nave, G., Nosek, B., Pfeiffer, T., Altmejd, A., Buttrick, N., Chan, T., Chen, Y., Forsell, E., Gampa, A., Heikenstein, E., Hummer, L., Imai, T., Isaksson, S., Manfredi, D., Rose, J., Wagenmakers, E., and Wu, H. (2018). Evaluating the replicability of social science experiments in Nature and Science between 2010 and 2015. *Nature Human Behavior*, 2:637 – 644.

Camerer, C. F., Dreber, A., Forsell, E., Ho, T., Huber, J., Johannesson, M., Kirchler, M., Almenberg, J., Altmejd, A., Chan, T., Heikensten, E., Holzmeister, F., Imai, T., Isaksson, S., Nave, G., Pfeiffer, T., Razen, M., and Wu, H. (2016). Evaluating replicability of laboratory experiments in economics. *Science*, 351:1433 – 1436.

Cooper, H., Hedges, V., and Valentine, J. C. (2009). *The Handbook of Research Synthesis and Meta-Analysis*. Russel Sage Foundation New York.

Cova, F., Strickland, B., Abatista, A., Allard, A., Andow, J., Attie, M., Beebe, J., Berniūnas, R., Boudesseul, J., Colombo, M., Cushman, F., Diaz, R., N'Djaye Nikolai van Dongen, N., Dranseika, V., Earp, B. D., Torres, A. G., Hannikainen, I., Hernández-Conde, J. V., Hu, W., Jaquet, F., Khalifa, K., Kim, H., Kneer, M., Knobe, J., Kurthy, M., Lantian, A., Liao, S.-y., Machery, E., Moerenhout, T., Mott, C., Phelan, M., Phillips, J., Rambharose, N., Reuter, K., Romero, F., Sousa, P., Sprenger, J., Thalabard, E., Tobia, K., Viciana, H., Wilkenfeld, D., and Zhou, X. (2018). Estimating the reproducibility of experimental philosophy. *Review of Philosophy and Psychology*.

Fisher, R. A. (1921). On the probable error of a coefficient of correlation deduced from a small sample. *Metron*, 1:3 – 32.

Open Science Collaboration (2015). Estimating the reproducibility of psychological science. *Science*, 349(6251):aac4716.
