# Supplementary material for: Probabilistic forecasting of replication studies
Source: PLoS One. 2020 Apr 22;15(4):e0231416. doi: 10.1371/journal.pone.0231416 (PMC7176099; doi:10.1371/journal.pone.0231416)
Supplement: S2 Appendix — Details and additional results. (PDF) [file pone.0231416.s002.pdf]

Probabilistic forecasting of replication studies
Supplement B: Supplementary results

Samuel Pawel, Leonhard Held

February 17, 2020

1 Sample size computations taking into account shrinkage and heterogeneity

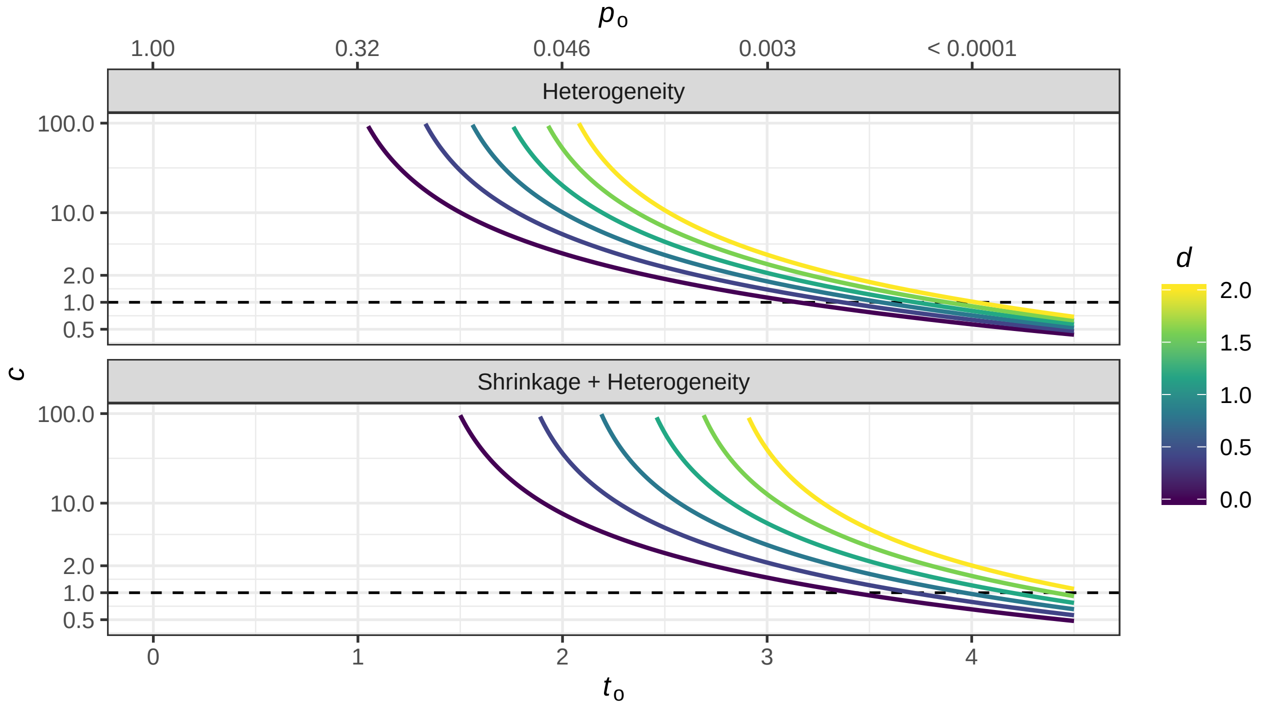

Figure 1: Required relative sample size $c = n_r/n_o$ to achieve a power of 80% as a function of the test statistic t_o (bottom axis) respectively the two-sided p-value p_o (top axis) of the original study and the relative between study heterogeneity $d = \tau^2/\sigma_o^2$.

Assuming that the standard errors of the effect estimates only depend on some unit variance κ^2 and the sample size of the study, *i. e.* $\sigma_o^2 = \kappa^2/n_o$ and $\sigma_r^2 = \kappa^2/n_r$, the required relative sample size $c = n_r/n_o$ to achieve a statistically significant result in the replication study with a certain power can be computed using root-finding algorithms. In Figure 1, the required c to achieve 80% power under the different models is shown as a function of t_o and the relative between study heterogeneity d. As can be seen, the required relative sample size c decreases for increasing t_o, *i. e.* evidence for an effect, and decreasing relative between study heterogeneity d. Furthermore, for small t_o, increasing d increases the required c much stronger than for large t_o. Comparing the shrinkage to the naive model, the required c under the shrinkage model is much larger for the same t_o, especially for small t_o. These results illustrate the fact that to achieve a reasonable power, the sample size of the replication study needs to be massively increased compared to the original study, when the results were only suggestive and/or subject to heterogeneity.

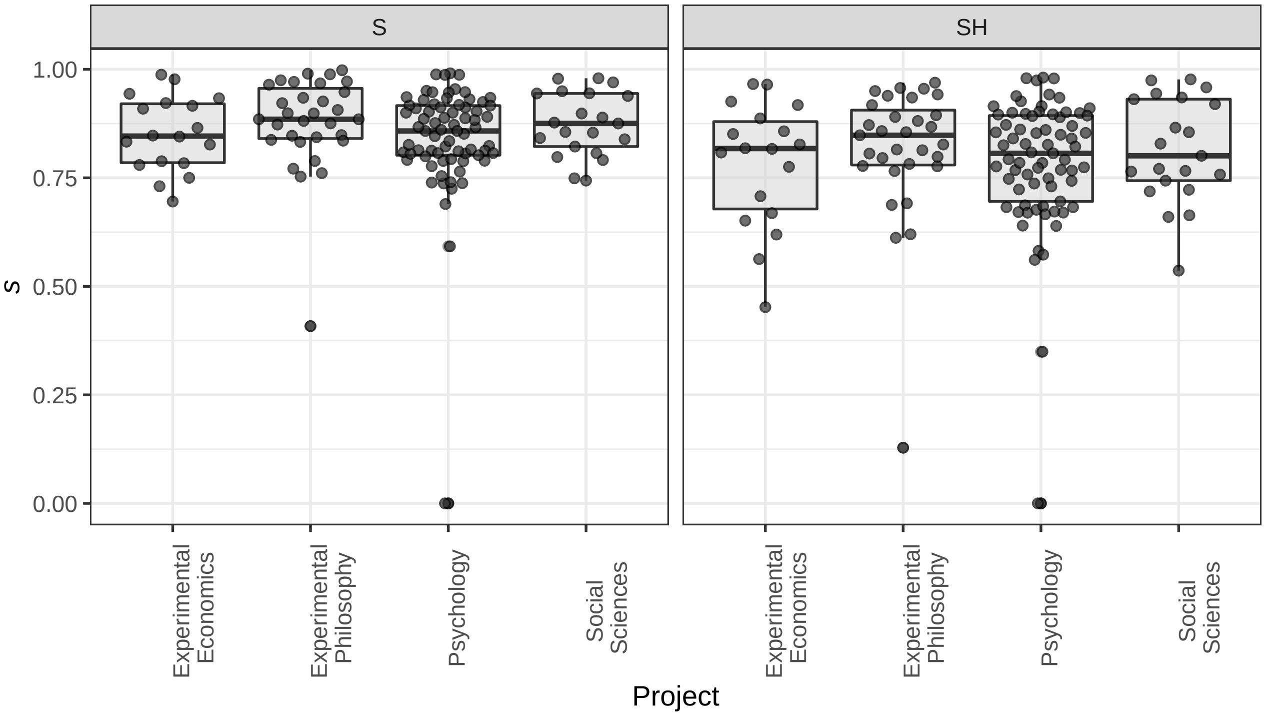

Figure 2: Obtained shrinkage factors s under S and SH method across replication projects.

2 Shrinkage across methods and projects

Figure 2 shows the shrinkage factors for the S forecasts (shrinkage method with $\tau = 0$) and for the SH forecasts (shrinkage method with $\tau = 0.08$). As expected, shrinkage under the SH method is larger than under the S method. However, there appears to be no large difference between the four projects with respect to the distribution of the shrinkage factors. The median shrinkage factor is about 0.8.

3 Forecasts of effect estimates

3.1 Testing for deviation from uniformity of PITs

Table 1 shows the results of Kolmogorov-Smirnov tests applied to the PIT values to test for miscalibration. In each data set, the test statistic of the SH method shows the smallest value, suggesting that there is the least evidence for this method to be miscalibrated. Looking at the economics data set, the tests provide weak evidence for miscalibration of the S and SH methods and moderate evidence for miscalibration of the N and H methods. In the philosophy data set, on the other hand, there is no evidence for miscalibration of any of the methods. Finally, in the social sciences and psychology data sets, the tests provide substantial evidence for miscalibration of all methods.

3.2 Scoring rules

Figure 3 shows the mean of the logarithmic scores (LS), continuous ranked probability scores (CRPS), and quadratic scores (QS) as well as their standard error for each data set and prediction method. It should be noted, that the standard errors are presented only to illustrate the spread of the individual scores and not to compare the mean scores between the different methods (this will be done further below using a paired test).

Table 2 shows the p-values of the paired Wilcoxon rank sum tests of the scores of the SH method compared to the scores of the other three prediction methods. Only these comparisons are reported since the SH method achieved the lowest mean score in all score types and data sets. For the forecasts in the psychology and social sciences data sets, there is in most cases strong evidence of a difference in scores between the SH method and the other methods. In the philosophy data set, on the other hand, there is no evidence for a difference between the scores of the SH method and the other methods. Finally, in the economics data set there is moderate evidence for a difference of the scores between the SH method and the N and H methods, however, no evidence for a difference of the scores between the SH and S methods.

Table 1: Kolmogorov-Smirnov tests comparing PIT values to $U(0,1)$ distribution.

Project	Method	Test statistic	p-value
Experimental Economics	N	0.38	0.007
$n = 18$	S	0.30	0.061
	H	0.39	0.006
	SH	0.29	0.073
Experimental Philosophy	N	0.21	0.11
$n = 31$	S	0.18	0.26
	H	0.19	0.20
	SH	0.08	0.97
Psychology	N	0.48	< 0.0001
$n = 73$	S	0.41	< 0.0001
	H	0.44	< 0.0001
	SH	0.36	< 0.0001
Social Sciences	N	0.61	< 0.0001
$n = 21$	S	0.52	< 0.0001
	H	0.54	< 0.0001
	SH	0.42	0.0008

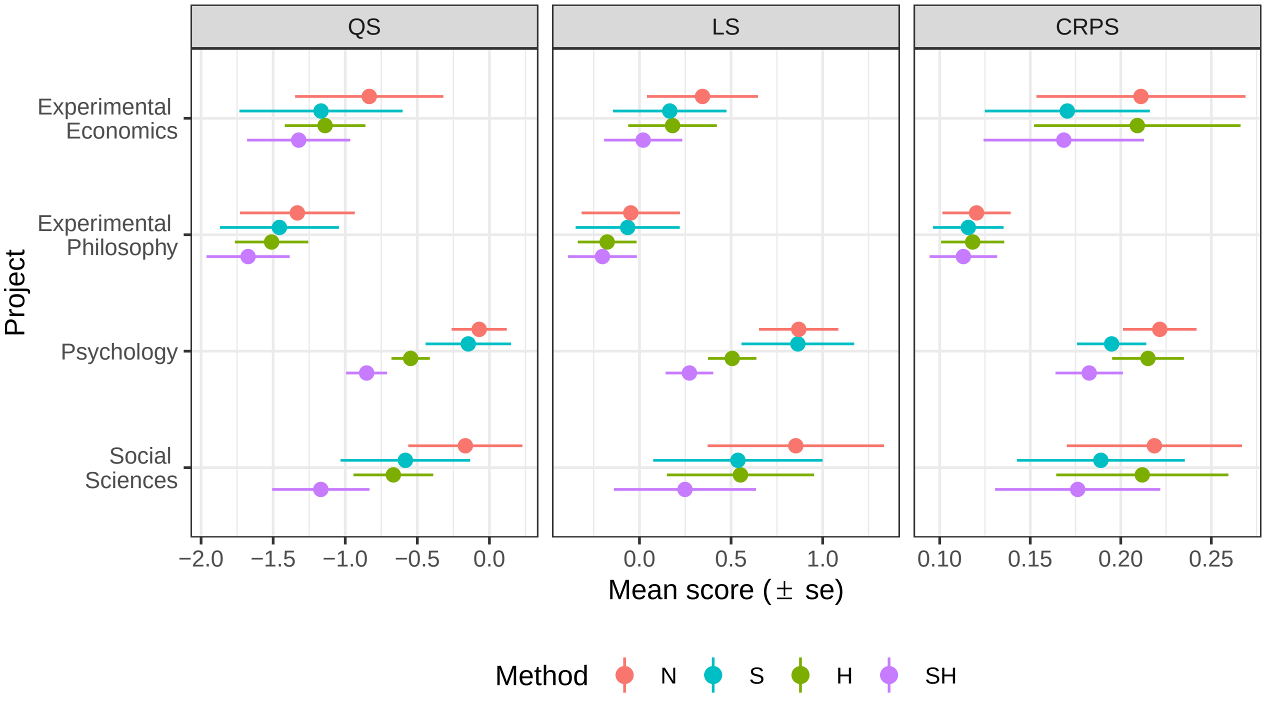

Figure 3: Mean scores with standard errors.

In Table 3, the results of the scoring rule based calibration tests are shown. The results of the four tests are often but not always in agreement. First, the unconditional test based on the logarithmic score suggests that all methods in the psychology and social sciences data sets are miscalibrated. The test also provides some evidence for miscalibration of the N and S methods in the economics and philosophy data sets. Second, the results from the unconditional test based on the CRPS provide evidence for miscalibration of all methods in the social sciences and psychology data sets, moderate evidence for miscalibration of the N method in the economics data set, and weak evidence for miscalibration of the N and S methods in the case of the philosophy data set. Third, the DSS regression test indicates miscalibration of all methods in the psychology data set and it provides weak evidence for miscalibration of the N method in the social sciences data set. Furthermore, the test does not suggest miscalibration of any method in the economics and philosophy data sets. Finally, the results from the CRPS regression test provide strong evidence for miscalibration of all methods in the psychology data set, no evidence for miscalibration of any method in the philosophy data sets, weak evidence for miscalibration of the N and S methods in the case of the economics data set, and weak evidence for miscalibration of all methods

Table 2: Results of paired Wilcoxon rank sum tests of the SH method vs. the other three methods.

Project	Test	Type QS p-value	LS p-value	CRPS p-value
Experimental Economics	N	0.038	0.016	0.007
$n = 18$	S	0.73	0.77	0.70
	H	0.014	0.005	0.003
Experimental Philosophy	N	0.44	0.95	0.66
$n = 31$	S	0.36	0.79	0.95
	H	0.26	0.28	0.47
Psychology	N	< 0.0001	< 0.0001	< 0.0001
$n = 73$	S	< 0.0001	< 0.0001	< 0.0001
	H	< 0.0001	< 0.0001	< 0.0001
Social Sciences	N	0.001	0.0007	0.0004
$n = 21$	S	0.018	0.07	0.001
	H	0.0003	< 0.0001	0.0005

except the SH method in the social sciences data set.

Table 3: Results from scoring rule based calibration tests.

		Test type							
		LS		CRPS		DSS-Regression		CRPS-Regression	
Project	Method	Statistic	p-value	Statistic	p-value	Statistic	p-value	Statistic	p-value
Experimental Economics	N	2.85	0.004	2.30	0.021	3.44	0.18	6.01	0.05
$n = 18$	S	2.11	0.035	1.23	0.22	3.40	0.18	7.01	0.03
	H	0.72	0.47	1.52	0.13	2.97	0.23	1.91	0.39
	SH	0.20	0.84	0.57	0.57	0.13	0.94	0.04	0.98
Experimental Philosophy	N	3.84	0.0001	2.02	0.044	3.67	0.16	3.98	0.14
$n = 31$	S	4.09	< 0.0001	2.13	0.033	3.55	0.17	3.79	0.15
	H	0.48	0.63	0.19	0.85	0.19	0.91	0.13	0.94
	SH	0.80	0.42	0.32	0.75	0.33	0.85	0.36	0.83
Psychology	N	12.86	< 0.0001	8.09	< 0.0001	31.13	< 0.0001	44.76	< 0.0001
$n = 73$	S	13.57	< 0.0001	6.68	< 0.0001	45.17	< 0.0001	80.35	< 0.0001
	H	6.25	< 0.0001	5.49	< 0.0001	16.48	0.0003	19.82	< 0.0001
	SH	4.28	< 0.0001	3.86	0.0001	8.18	0.017	9.56	0.008
Social Sciences	N	7.68	< 0.0001	6.02	< 0.0001	6.26	0.044	9.39	0.009
$n = 21$	S	6.00	< 0.0001	4.86	< 0.0001	4.49	0.11	6.14	0.046
	H	4.30	< 0.0001	4.03	< 0.0001	3.81	0.15	5.11	0.078
	SH	2.79	0.005	2.80	0.005	2.88	0.24	3.33	0.19

4 Forecasts of statistical significance

4.1 Estimated probability of statistical significance

Figure 4 shows the probabilities of a statistically significant test statistic in the replication study under the investigated predictive distributions, grouped by whether or not the replications actually achieved significance. When looking at the statistical methods, the estimated probabilities of significance are generally high, even for many of the studies where the replications did not achieve significance. Comparing the different replication

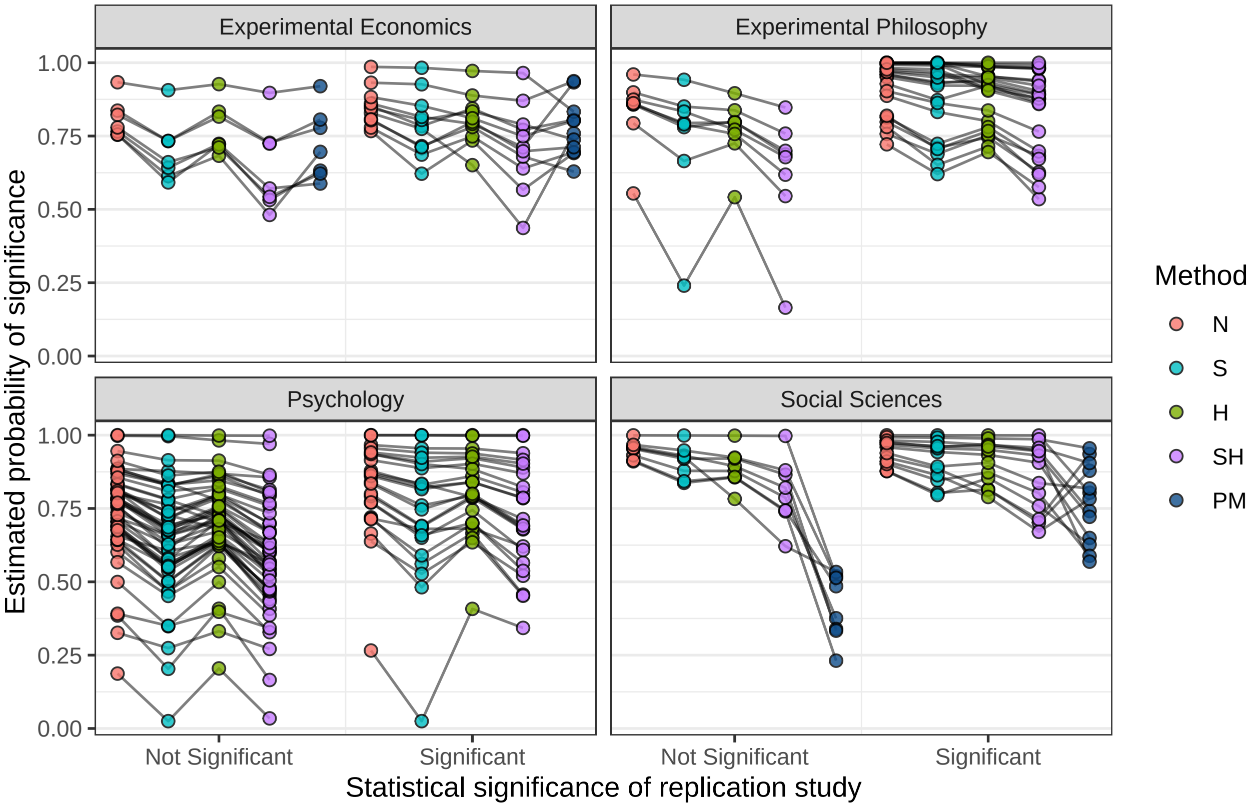

Figure 4: Probabilities of statistically significant replication outcome under predictive distributions (at $\alpha = 0.05$).

projects, in the social science data set the distributions of the estimated probabilities among all methods are virtually identical between the significant and non-significant replication studies, suggesting low discriminatory power of all methods. In the economics, philosophy, and psychology data sets, on the other hand, the estimated probabilities of the non-significant replications are in most cases slightly smaller, indicating some discriminatory power of the forecasts. Looking at the different prediction methods, the estimated probabilities are generally smaller for the S compared to the N method, and similarly for the H compared to SH method. Moreover, in the social sciences data set the probabilities from the non-statistical prediction market method are much lower for non-significant replications compared to the probabilities of the significant replications, suggesting substantial discriminatory power of this method. In the economics data set, however, the prediction market probabilities are high for both significant and non-significant replications, indicating only low discriminatory power.

4.2 Expected vs. observed number of significant replications at different thresholds

It is also interesting to compare the expected and observed number of statistically significant replication outcomes for smaller significance thresholds than 0.05, as shown in Figure 5. For all values of α, the expected number is smaller for the S and SH methods than for the N and H methods, and it is also smaller when taking into account heterogeneity compared to when not taking heterogeneity into account. These results indicate again that the SH method leads to more realistic forecasts. Comparing the different data sets, in the psychology and social sciences data sets the difference between the expected and observed number of significant replications is large across the whole range of possible significance thresholds for all four prediction methods. In the philosophy and the economics data set, on the other hand, the expected number is much closer to the observed

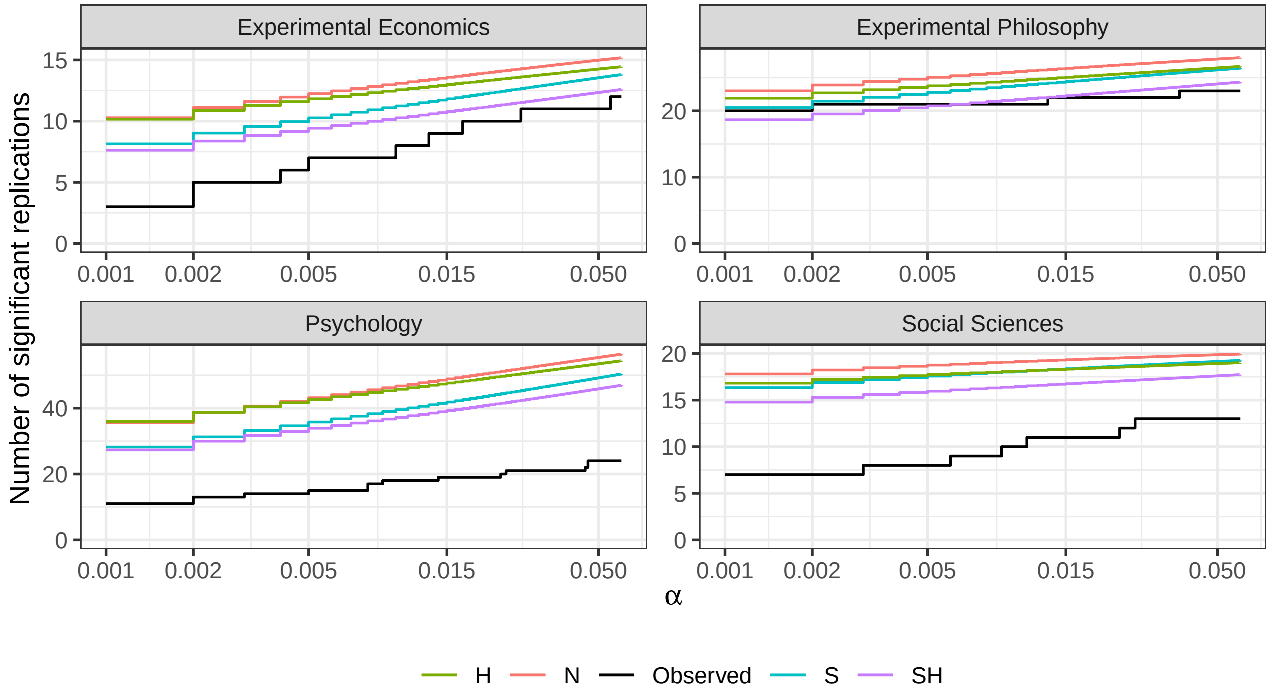

Figure 5: Expected and observed number of statistically significant replication studies as function of α.

number, especially for the S and SH methods.

4.3 Brier scores

In Figure 6 the mean Brier scores are shown visually with the corresponding standard errors. Note that the standard errors are only shown to illustrate the spread of the individual scores and not to compare the mean scores between the methods.

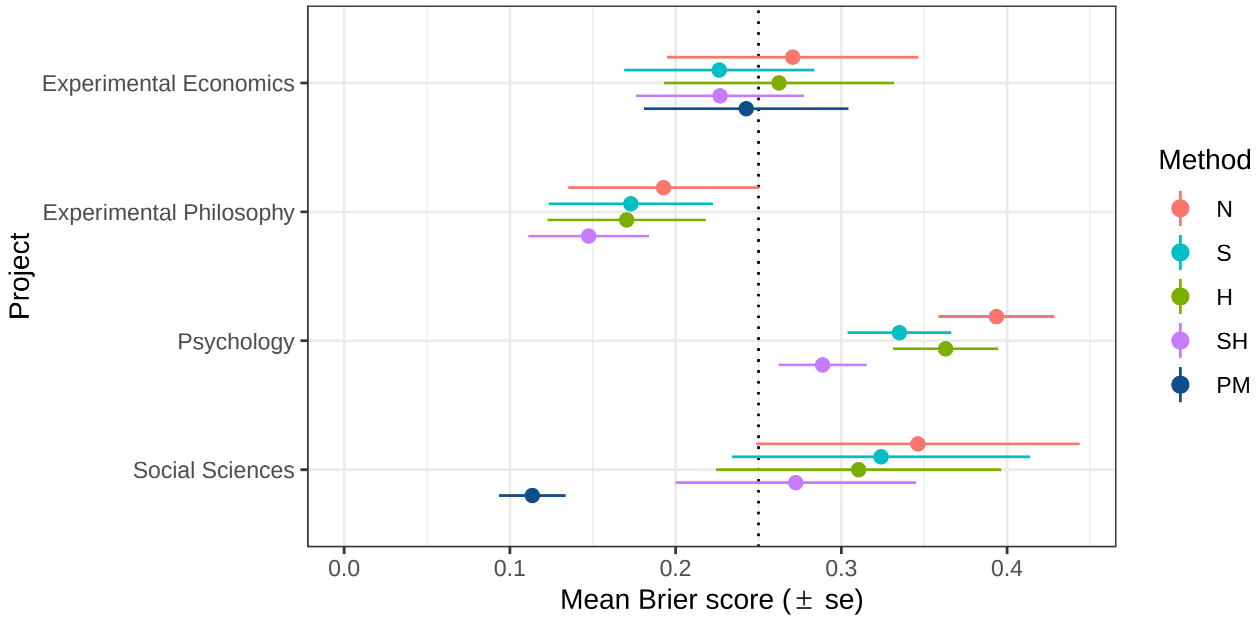

Figure 6: Mean Brier scores.

The mean Brier scores were also computed for binary forecasts at other significance thresholds α than 0.05, as shown in Figure 7. Across the whole range of α values, the SH method shows the smallest mean Brier scores in all data sets, while the N method usually shows the largest mean Brier score.

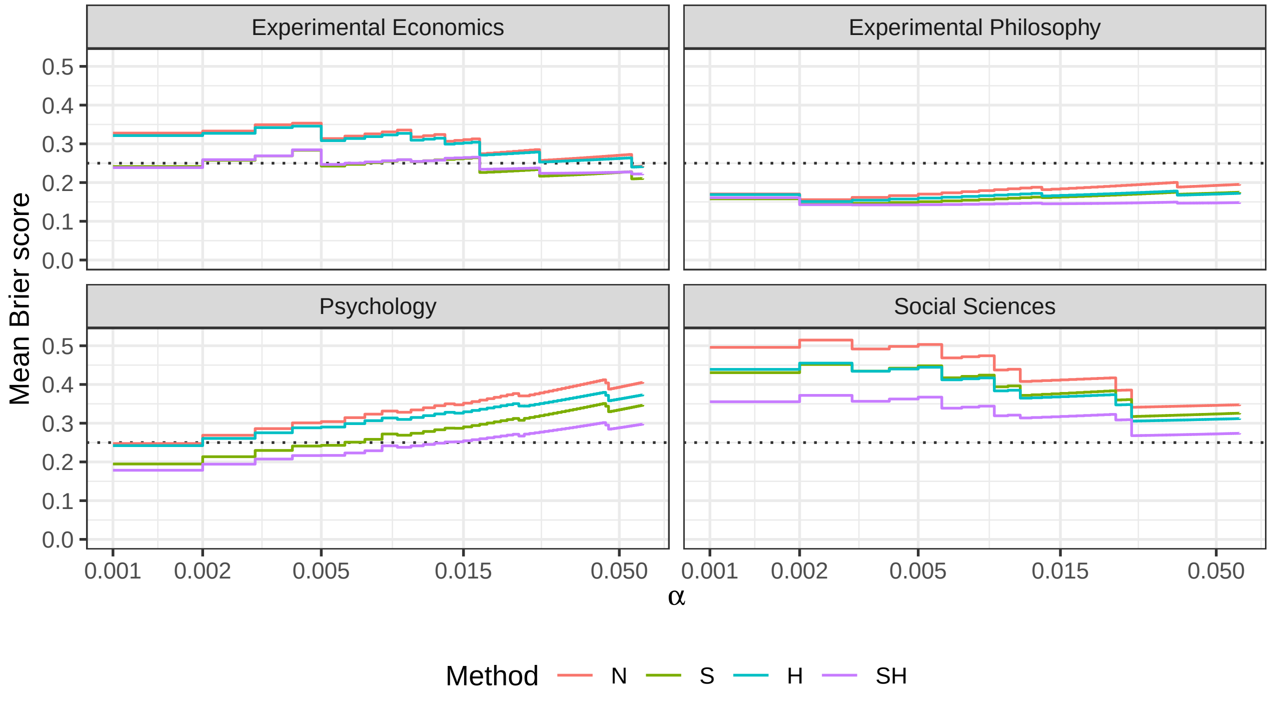

Figure 7: Mean Brier score as function of α.

4.4 Calibration slope

Figure 8 shows the calibration slopes of the statistical forecasts for smaller significance thresholds α than 0.05. Looking at the psychology and social sciences data sets, all calibration slopes increase slightly for lower values

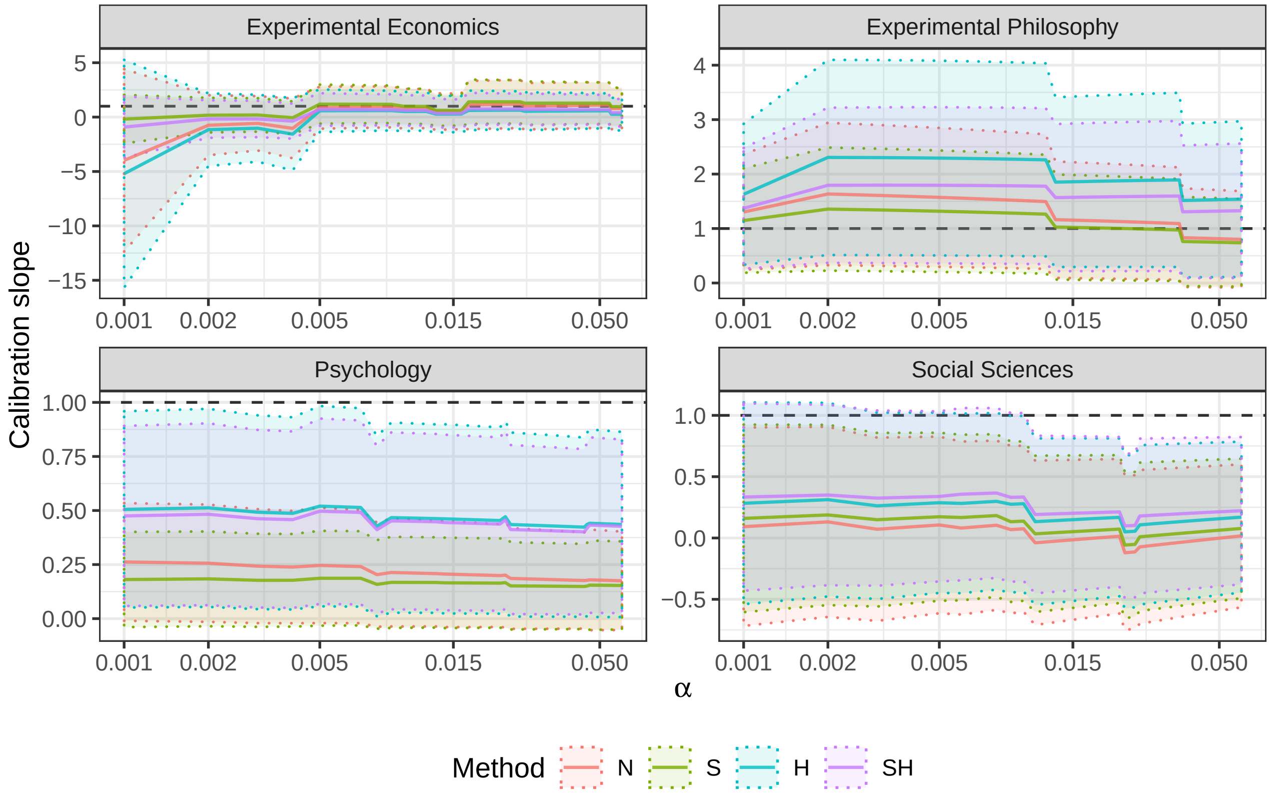

Figure 8: Calibration slopes with 95% confidence intervals as function of α.

of α, with the H and SH methods showing the highest values, yet all still remain below the nominal value of

one. In the the economics data set, one the other hand, the calibration slopes become smaller with decreasing α for all methods, the N and H methods show even negative values. Furthermore, for decreasing values of α the confidence intervals of the calibration slope become extremely wide. Finally, in the philosophy data set the slopes of all methods increase with decreasing α until $\alpha = 0.002$, where they start to decrease again to values close to one.

4.5 Area under the curve

Figure 9 shows the AUC as a function of the significance threshold α. Due to fewer replications that are

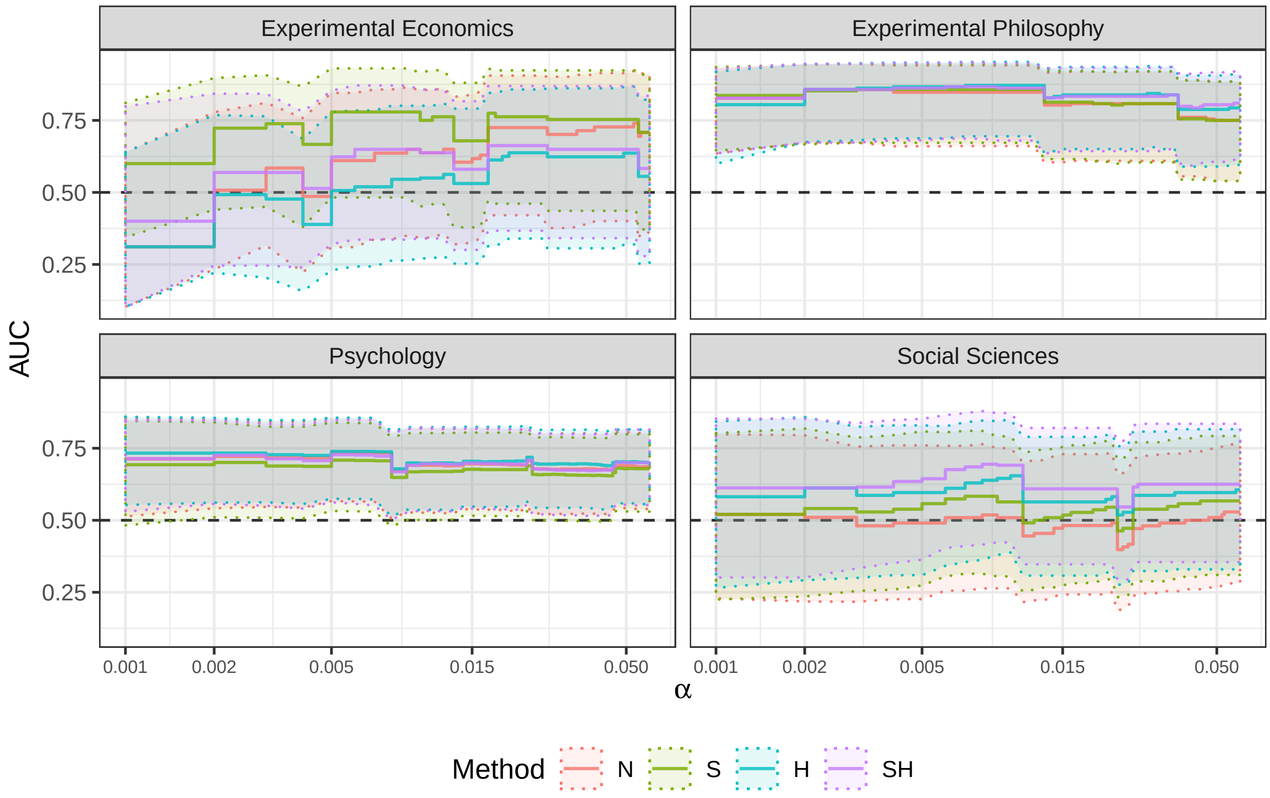

Figure 9: Area under the curve with 95% confidence intervals as function of α.

significant for small α, the confidence intervals become wider as α decreases. In the philosophy, social sciences, and psychology data sets, the AUCs of all methods stay more or less constant over the entire range of α. Namely, the AUCs of all methods remain around 0.5 to 0.6 in the social sciences data set, while they remain around 0.7 to 0.8 in the psychology and philosophy data sets. In the economics data set, on the other hand, for all methods the AUCs also decrease with decreasing α to values of around 0.5.

5 Comparing default heterogeneity value with empirical distribution of estimates

Figure 10 shows a histogram of 497 between-study heterogeneity estimates from meta-analyses with correlation effect sizes published in the journal *Psycholgical Bulletin* between 1990 and 2013 (Erp et al., 2017). The dashed line indicates the chosen default value of $\tau = 0.08$ which corresponds to the 34% quantile of this distribution. This seems reasonable to us, as those estimates stem from meta-analysis of ordinary studies that are likely to be more heterogeneous than direct replication studies, yet the value of 0.08 is still sufficiently different from zero such that we can investigate whether predictive performance can be improved compared to forecasts not taking into account heterogeneity.

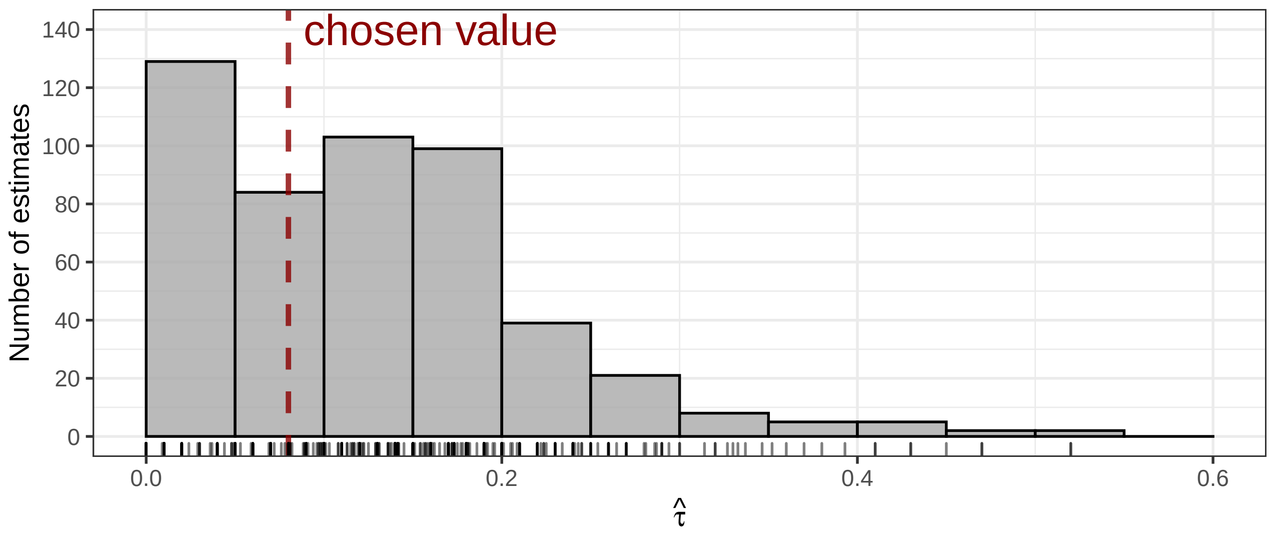

Figure 10: Empirical distribution of 497 between-study heterogeneity estimates of meta-analyses with correlation effect sizes in the journal *Psycholgical Bulletin* between 1990 and 2013 (Erp et al., 2017). Chosen default value of $\tau = 0.08$ for forecasts taking into account heterogeneity indicated by dashed line.

References

Erp, S. V., Verhagen, J., Grasman, R. P. P. P., and Wagenmakers, E.-J. (2017). Estimates of between-study heterogeneity for 705 meta-analyses reported in *psychological bulletin* from 1990-2013. *Journal of Open Psychology Data*, 5(1):4.
