# Supplementary material for: Probabilistic forecasting of replication studies
Source: PLoS One. 2020 Apr 22;15(4):e0231416. doi: 10.1371/journal.pone.0231416 (PMC7176099; doi:10.1371/journal.pone.0231416)
Supplement: S3 Appendix — Code to reproduce analyses, plots, and tables. (PDF) [file pone.0231416.s003.pdf]

Probabilistic forecasting of replication studies
Supplement C: Code

Samuel Pawel, Leonhard Held

February 25, 2020

Data, packages, global options

```
# Packages
library("dplyr")
library("tidyr")
library("ggplot2")
library("ggbeeswarm")
library("ggpubr")
library("tables")
library("biostatUZH")
# can be installed with: install.packages("biostatUZH", repos = "http://R-Forge.R-project.org")

# Load meta-analytic subset data (i.e. SE of Fisher z-transformed correlation can be computed)
replication_data <- read.csv("../../Data/replications_ma_subset.csv")

# Prediction market data
pm_data <- replication_data %>%
  filter(!is.na(Market_Belief))

# Levels and labels of factors
levels_projects <- c("Experimental Economics",
                     "Experimental Philosophy",
                     "Psychology",
                     "Social Sciences")
labels_projects2lines <- c("Experimental \nEconomics",
                           "Experimental \nPhilosophy",
                           "Psychology",
                           "Social \nSciences")
levels_methods <- c("N",
                    "S",
                    "H",
                    "SH")
levels_methods_pm <- c(levels_methods, "PM")
levels_scores <- c("QS",
                   "LS",
                   "CRPS")

# Colors
colors_methods <- c("N" = "#F8766D",
                    "H" = "#7CAE00",
                    "S" = "#00BFC4",
                    "SH" = "#C77CFF",
                    "PM" = "#104E8B")
```

Figure 1

```r
# Compute summary statistics
summaries_data <- replication_data %>%
  group_by(Project) %>%
  summarise(mean_r_OS = round(mean(r_OS), 2),
            mean_r_RS = round(mean(r_RS), 2),
            perc_significant = 100*round(mean(pval_RS < 0.05), 2))

# Plot of r_o vs r_r
ggplot(data = replication_data, aes(x = r_OS, y = r_RS)) +
  geom_hline(yintercept = 0, lty = 2) +
  geom_abline(intercept = 0, slope = 1, col = "grey") +
  geom_rug(aes(col = pval_RS_significant), alpha = 0.8) +
  geom_point(aes(fill = pval_RS_significant), alpha = 0.8, size = 2, shape = 21) +
  geom_text(data = summaries_data, parse = TRUE, size = 5,
            aes(x = -0.1, y = 0.9, label = paste("bar(italic(r))[o] ==~", mean_r_OS))) +
  geom_text(data = summaries_data, parse = TRUE, size = 5,
            aes(x = -0.1, y = 0.65, label = paste("bar(italic(r))[r] ==~", mean_r_RS))) +
  geom_text(data = summaries_data, parse = FALSE, size = 5,
            aes(x = 0.12, y = -0.35, label = paste(perc_significant, "% significant", sep = ""))) +
  facet_wrap(~ Project) +
  labs(x = expression(paste("Original effect estimate (", italic(r), ")")),
       y = expression(paste("Replication effect estimate (", italic(r), ")"))) +
  lims(x = c(-0.5, 1), y = c(-0.5, 1)) +
  guides(fill = guide_legend(title = expression(paste("Replication ", italic("p"), "-value"))),
         color = FALSE) +
  coord_fixed() +
  theme_bw()
```

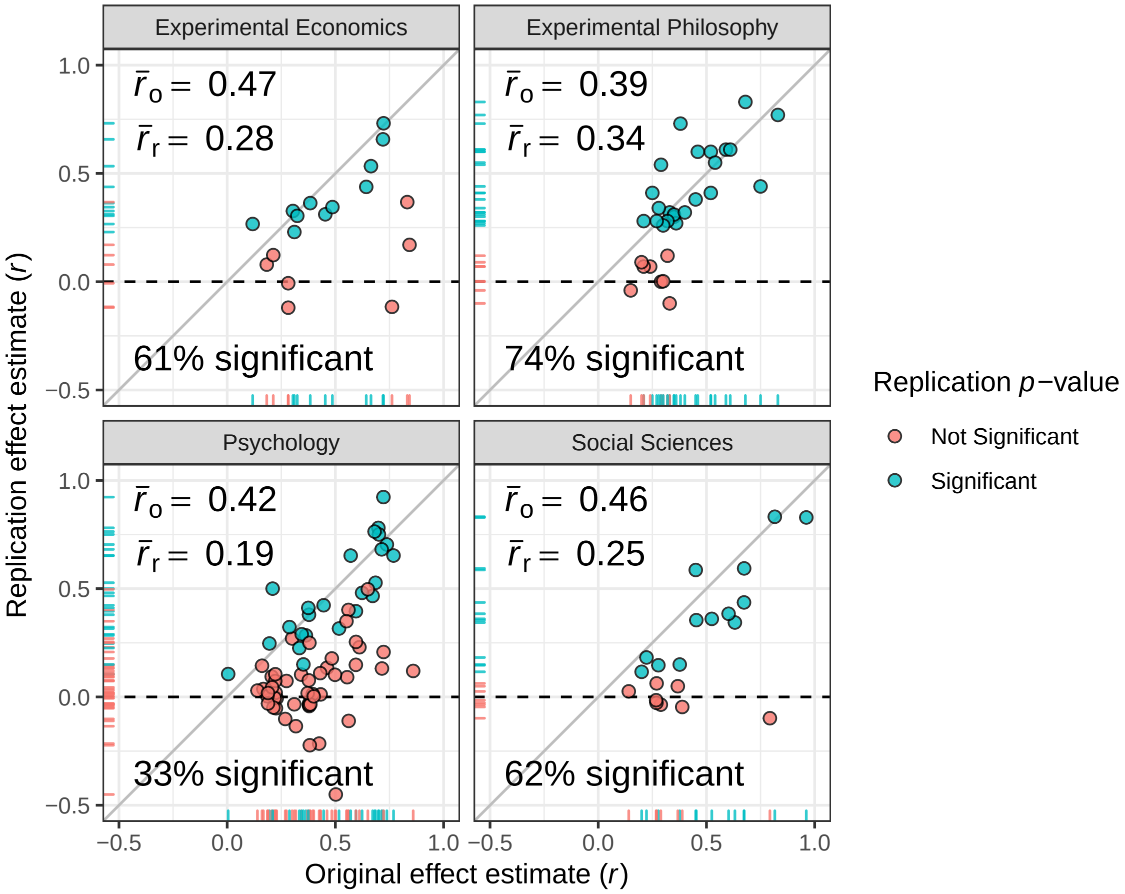

Figure 2

```r
# Plot significance of replication vs. prediction market beliefs
ggplot(data = pm_data, aes(x = pval_RS_significant, y = Market_Belief)) +
  geom_boxplot(alpha = 0.5, fill = "lightgrey") +
  geom_quasirandom(size = 2, shape = 21, alpha = 0.9, fill = "grey20") +
  facet_wrap(~ Project) +
  labs(x = expression(paste("Replication ", italic(p), "-value")),
       y = "Prediction market belief") +
  lims(y = c(0, 1)) +
  theme_bw()
```

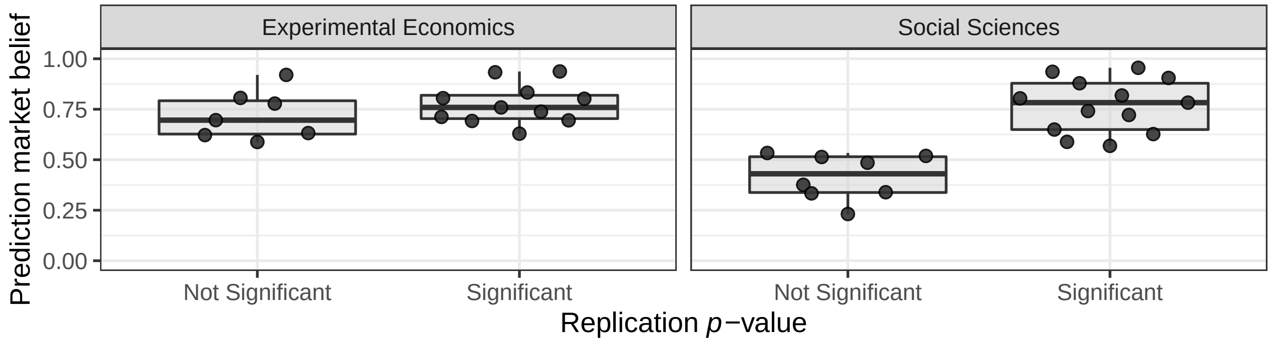

Figure 4

```r
# Plot showing shrinkage factor as function of t_o and d
shrinkage_factor <- function(t_o, d) pmax(1 - (1 + d)/t_o^2, 0)
parameters <- expand.grid(t_o = seq(0, 4.5, 0.01),
                          d = seq(0, 2, 0.4))
s <- apply(parameters, 1, function(par) shrinkage_factor(t_o = par[1], d = par[2]))
shrinkage_data_df <- data.frame(parameters, s = s)

z_to_p <- function(z) 2*pnorm(z, lower.tail = FALSE)
pval_labels <- formatPval(z_to_p(z = seq(0, 4, 1)))

ggplot(data = shrinkage_data_df, aes(x = t_o, y = s, color = d, group = factor(d))) +
  geom_line(size = 0.8, alpha = 0.8) +
  labs(x = expression(italic(t)[o]), y = expression(italic(s))) +
  guides(color = guide_colorbar(title = expression(~~italic(d)))) +
  scale_color_viridis_c() +
  scale_x_continuous(sec.axis = sec_axis(trans = ~z_to_p(.), breaks = z_to_p(seq(0, 4, 1)),
                                         labels = pval_labels, name = expression(italic(p)[o])),
                     breaks = seq(0, 4, 1))  +
  ylim(0, 1) +
  theme_bw()
```

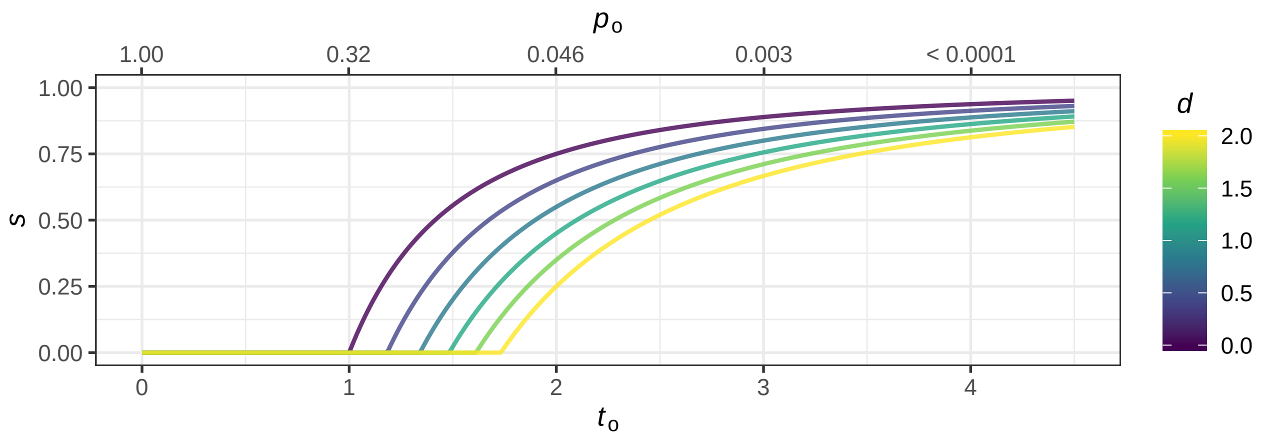

Figure 5

```
# Function to compute predictive density/cdf
predict_t_r <- function(x, t_o, c, d, shrinkage = FALSE) {
  if (shrinkage == TRUE) s <- pmax(1 - (1 + d)/t_o^2, 0)
  else s <- 1
  mu <- s*t_o*sqrt(c)
  sigma <- sqrt(s*(c + d*c) + 1 + d*c)
  return(pnorm(q = x, mean = mu, sd = sigma, lower.tail = FALSE))
}

# Apply function to parameter grid
parameters <- expand.grid(t_o = seq(0, 4.5, 0.01),
                          c = c(0.5, 1, 2, 4),
                          d = c(0, 1),
                          shrinkage = c(FALSE, TRUE))
probabilities <- apply(parameters, 1, function(par) {
  power <- predict_t_r(x = qnorm(0.975), t_o = par[1], c = par[2], d = par[3], shrinkage = par[4])
  data.frame(power = power, t_o = par[1], c = par[2], d = par[3], shrinkage = par[4])
}) %>%
  bind_rows() %>%
  mutate(Method = case_when(d == 0 & shrinkage == 0 ~ "N",
                            d == 0 & shrinkage == 1 ~ "S",
                            d == 1 & shrinkage == 0 ~ "H",
                            d == 1 & shrinkage == 1 ~ "SH"),
         Method = factor(Method, levels = levels_methods,
                         labels = c("Naive", "Shrinkage", "Heterogeneity",
                                    "Shrinkage + Heterogeneity")))
colors_fig5 <- c("Naive" = "#F8766D",
                 "Heterogeneity" = "#7CAE00",
                 "Shrinkage" = "#00BFC4",
                 "Shrinkage + Heterogeneity" = "#C77CFF")
# Plot of estimated probabilities of stat. significance in replication
pval_labels <- formatPval(z_to_p(seq(0, 4, 1)))
ggplot(data = probabilities, aes(x = t_o, y = power, color = Method)) +
  geom_vline(xintercept = qnorm(0.975), lty = 2, alpha = 0.8) +
  geom_line(alpha = 0.9, size = 0.8) +
  labs(x = expression(italic(t[o])), y = "Pr(Replication significant)") +
  facet_wrap(~ c, ncol = 2, labeller = label_bquote(italic(c) ==~ .(round(c, 2)))) +
    scale_x_continuous(sec.axis = sec_axis(trans = ~z_to_p(.), breaks = z_to_p(seq(0, 4, 1)),
                                           labels = pval_labels, name = expression(italic(p)[o])),
                       breaks = seq(0, 5, 1))  +
  scale_color_manual(values = colors_fig5) +
  theme_bw() +
  theme(legend.position = "bottom")
```

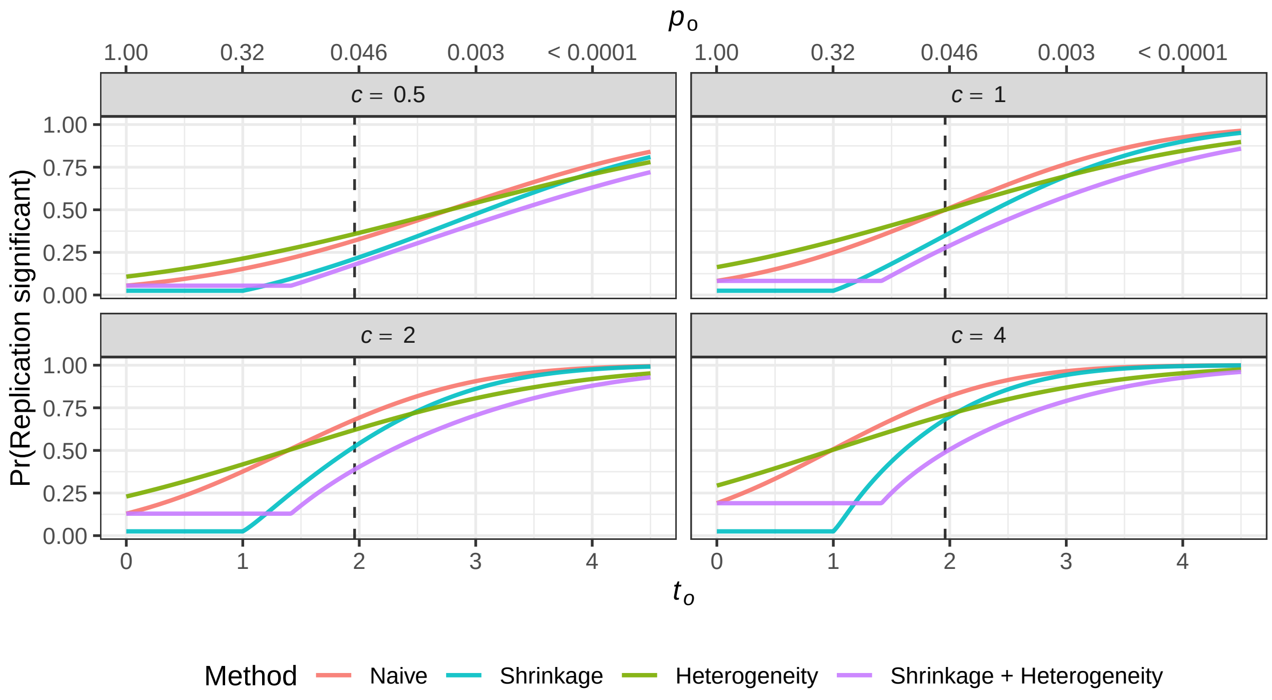

Figure 6

```
quantile_difference <- function(tau, lower = 0.025, upper = 0.975) {
  # for given heterogeneity tau, returns difference of quantiles upper - lower
  # after transformation with tanh
  l <- qnorm(lower)
  u <- qnorm(upper)
  corr_diff <- tanh(tau*u) - tanh(tau*l)
  return(corr_diff)
}
find_tau_difference <- function(diff, lower = 0.025, upper = 0.975) {
  root_fun <- function(t) quantile_difference(tau = t, lower = lower, upper = upper) - diff
  tau <- uniroot(f = root_fun, lower = 0, upper = 100)$root
  return(tau)
}
r_classification <- data.frame(size = c("small", "medium", "large"),
                               r = c(0.1, 0.3, 0.5))
r_classification$tau <- sapply(r_classification$r, function(r) find_tau_difference(diff = r))

# Plot of relationship between tau hyperparameter and difference of 97.5% to 2.5% quantile
# of backtransformed correlations r = tanh(theta_k)
taus <- seq(0, 0.7, 0.01)
tau_difference <- data.frame(tau = taus,
                             diff = sapply(taus, quantile_difference))
ggplot(data = tau_difference, aes(x = tau, y = diff)) +
  geom_line() +
  geom_segment(data = r_classification, aes(x =  tau, xend = tau, y = 0, yend = r), lty = 3) +
  geom_segment(data = r_classification, aes(x =  0, xend = tau, y = r, yend = r), lty = 3) +
  geom_text(data = r_classification, aes(x = -0.03, y = r, label = size), size = 3) +
  labs(x = bquote(tau), y = expression(delta(tau))) +
  scale_x_continuous(breaks = c(0, round(r_classification$tau, 2), seq(0.25, 1, 0.25)),
                     labels = c("0", round(r_classification$tau, 2), seq(0.25, 1, 0.25)),
                     minor_breaks = NULL) +
  # coord_flip() +
  theme_bw()
```

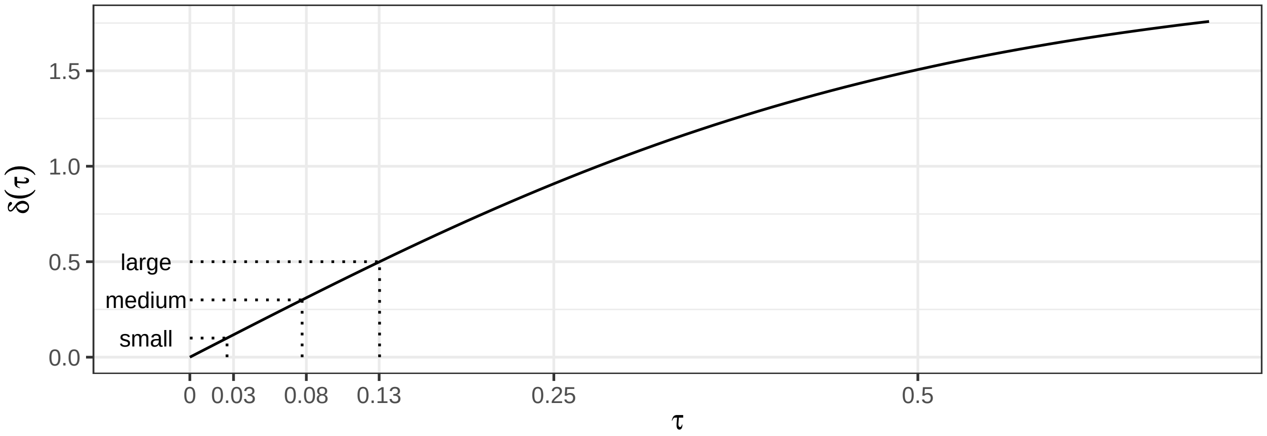

Helper functions to obtain predictive distributions

```
# Default value for tau hyperparameter (see article for how this was determined)
tau_default <- 0.08

# Functions to obtain parameters of predictive distributions
get_params <- function(theta_o, sigma_o, sigma_r, tau = tau_default, prior = "flat") {
  t_o <- theta_o/sigma_o
  d <- tau^2/sigma_o^2
  if (prior == "sceptical") s <- pmax(1 - (1 + d)/t_o^2, 0)
  if (prior == "flat") s <- 1
  mu <- s*theta_o
  sigma <- sqrt(s*(sigma_o^2 + tau^2) + sigma_r^2 + tau^2)
  return(data.frame("mu" = mu, "sigma" = sigma))
}

prediction_methods <- list(
  "N" = function(theta_o, sigma_o, sigma_r, tau) {
    get_params(theta_o, sigma_o, sigma_r, tau = 0, prior = "flat")
    },
  "S" = function(theta_o, sigma_o, sigma_r, tau) {
    get_params(theta_o, sigma_o, sigma_r, tau = 0, prior = "sceptical")
    },
  "H" = function(theta_o, sigma_o, sigma_r, tau) {
    get_params(theta_o, sigma_o, sigma_r, tau = tau, prior = "flat")
    },
  "SH" = function(theta_o, sigma_o, sigma_r, tau) {
    get_params(theta_o, sigma_o, sigma_r, tau = tau, prior = "sceptical")
    }
  )

get_params_all_methods <- function(theta_o, sigma_o, sigma_r, tau = tau_default,
                                   methods = prediction_methods) {
  params <- lapply(methods, function(f) f(theta_o = theta_o, sigma_o = sigma_o,
                                          sigma_r = sigma_r, tau = tau))
  return(params)
}
```

Figure 7

```r
# Helper function to compute prediction intervals for all methods
get_PIs <- function(theta_o, sigma_o, sigma_r, tau = tau_default, gamma = 0.95) {
  params <- get_params_all_methods(theta_o = theta_o, sigma_o = sigma_o, sigma_r = sigma_r, tau = tau)
  result <- lapply(seq_along(params), function(i) {
    data.frame("PI_lower" = qnorm(p = (1 - gamma)/2, mean = params[[i]]$mu, sd = params[[i]]$sigma),
               "PI_upper" = qnorm(p = (1 + gamma)/2, mean = params[[i]]$mu, sd = params[[i]]$sigma),
               "Method" = names(params)[i])
  })
  result_df <- do.call(rbind, result)
  return(result_df)
}

# Compute 95% prediction intervals
PI <- with(replication_data, get_PIs(theta_o = FZ_OS, sigma_o = FZ_se_OS, sigma_r = FZ_se_RS))
PI_df <- data.frame(PI, replication_data) %>%
  mutate(Method = factor(Method, levels = levels_methods),
         within = (FZ_RS > PI_lower) & (FZ_RS < PI_upper),
         within = factor(within, labels = c("Outside prediction interval",
                                            "Within prediction interval")),
         PI_lower_r = tanh(PI_lower),
         PI_upper_r = tanh(PI_upper))

# Compute coverage
summary_PI <- PI_df %>%
  group_by(Project, Method) %>%
  summarise(prop_within = round(mean(within == "Within prediction interval"), 2),
            prop_within = paste(prop_within*100, "% coverage", sep = ""))

# Plot of r_o vs r_r showing 95% prediction intervals and coverage
ggplot(PI_df, aes(x = r_OS, y = r_RS)) +
  geom_hline(yintercept = 0, lty = 2) +
  geom_abline(intercept = 0, slope = 1, lty = 1) +
  geom_pointrange(aes(ymin = PI_lower_r, ymax = PI_upper_r, col = within), size = 0.6,
                  alpha = 0.8, fatten = 1.5, key_glyph = "point") +
  geom_point(alpha = 0.5, size = 0.3) +
  geom_text(data = summary_PI, aes(x = 0.35, y = 0.93, label = prop_within), size = 4) +
  labs(x = expression(paste("Original effect estimate (", italic(r), ")")),
       y = expression(paste("Replication effect estimate (", italic(r), ")"))) +
  scale_x_continuous(breaks = c(0, 0.5, 1), limits = c(0, 1)) +
  scale_color_manual(within, values = c("Outside prediction interval" = "darkred",
                                        "Within prediction interval" = "gray60")) +
  facet_grid(Project ~ Method) +
  theme_bw() +
  theme(strip.text = element_text(size = 8), legend.position = "bottom") +
  guides(color = guide_legend(title = NULL))
```

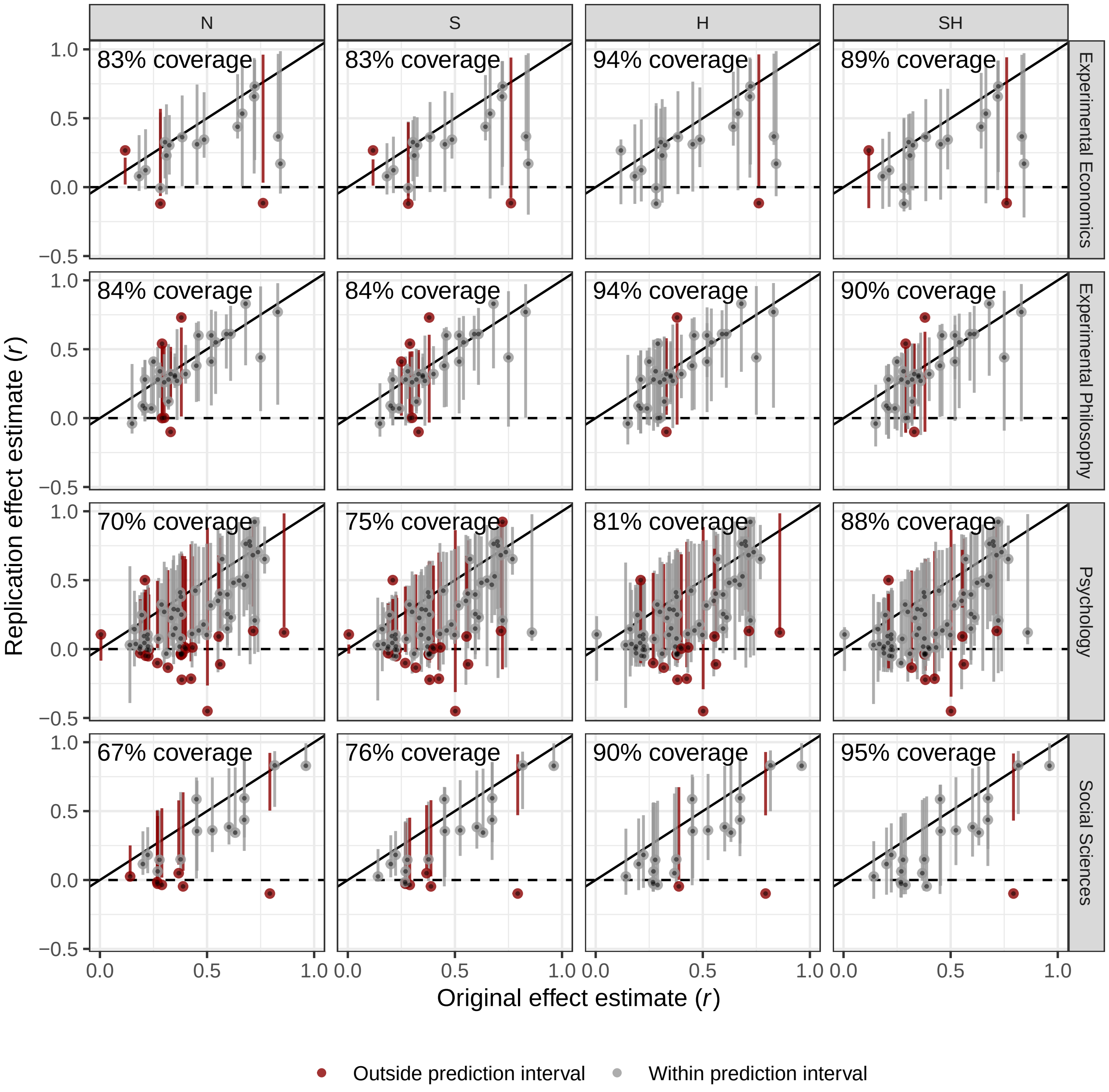

Figure 8

```r
# Helper function to compute PIT
get_PITs <- function(theta_o, theta_r, sigma_o, sigma_r, tau = tau_default) {
  params <- get_params_all_methods(theta_o = theta_o, sigma_o = sigma_o,
                                   sigma_r = sigma_r, tau = tau)
  PITs <- lapply(seq_along(params), function(i) {
    data.frame("PIT" = pnorm(q = theta_r, mean = params[[i]]$mu, sd = params[[i]]$sigma),
               "Method" = names(params)[i])
  })
  PITs_df <- do.call(rbind, PITs)
  return(PITs_df)
}

# Compute PITs
PITs <- with(replication_data, get_PITs(theta_o = FZ_OS, theta_r = FZ_RS,
                                        sigma_o = FZ_se_OS, sigma_r = FZ_se_RS))
PIT_data <- cbind(replication_data, PITs)
```

```r
# Test with Kolmogorov Smirnov test whether PIT values deviate from U(0, 1)
options(scipen = 5)
n_breaks <- 8
PIT_ks <- PIT_data %>%
  group_by(Project, Method) %>%
  summarise(t = ks.test(PIT, y = "punif", 0, 1, exact = TRUE)$statistic,
            pvalue = ks.test(PIT, y = "punif", 0, 1, exact = TRUE)$p.value,
            Test = "KS",
            uniform_count = n()/n_breaks) %>%
  ungroup()
PIT_ks_plot <- PIT_ks %>%
  mutate(pstring = ifelse(pvalue < 0.0001, "italic(p)", "italic(p) == ~"),
         pvalue = paste(pstring, biostatUZH::formatPval(pvalue)))

# Plot histograms of PITs
ggplot(data = PIT_data, aes(x = PIT)) +
  geom_histogram(breaks = seq(0, 1, length.out = n_breaks), col = 1, fill = "darkgrey") +
  geom_text(data = PIT_ks_plot, aes(x = Inf, y = Inf, label = pvalue),
            hjust = 1.1, vjust = 1.3, parse = TRUE) +
  geom_hline(data = PIT_ks_plot, aes(yintercept = uniform_count), lty = 2, alpha = 0.7) +
  facet_grid(Project ~ Method, scales = "free_y") +
  labs(x = "PIT", y = "Count") +
  scale_x_continuous(breaks = seq(0, 1, 0.25), labels = c("0.0", seq(0.25, 0.75, 0.25), "1.0")) +
  theme_bw() +
  theme(strip.text.x = element_text(size = 8), strip.text.y = element_text(size = 6))
```

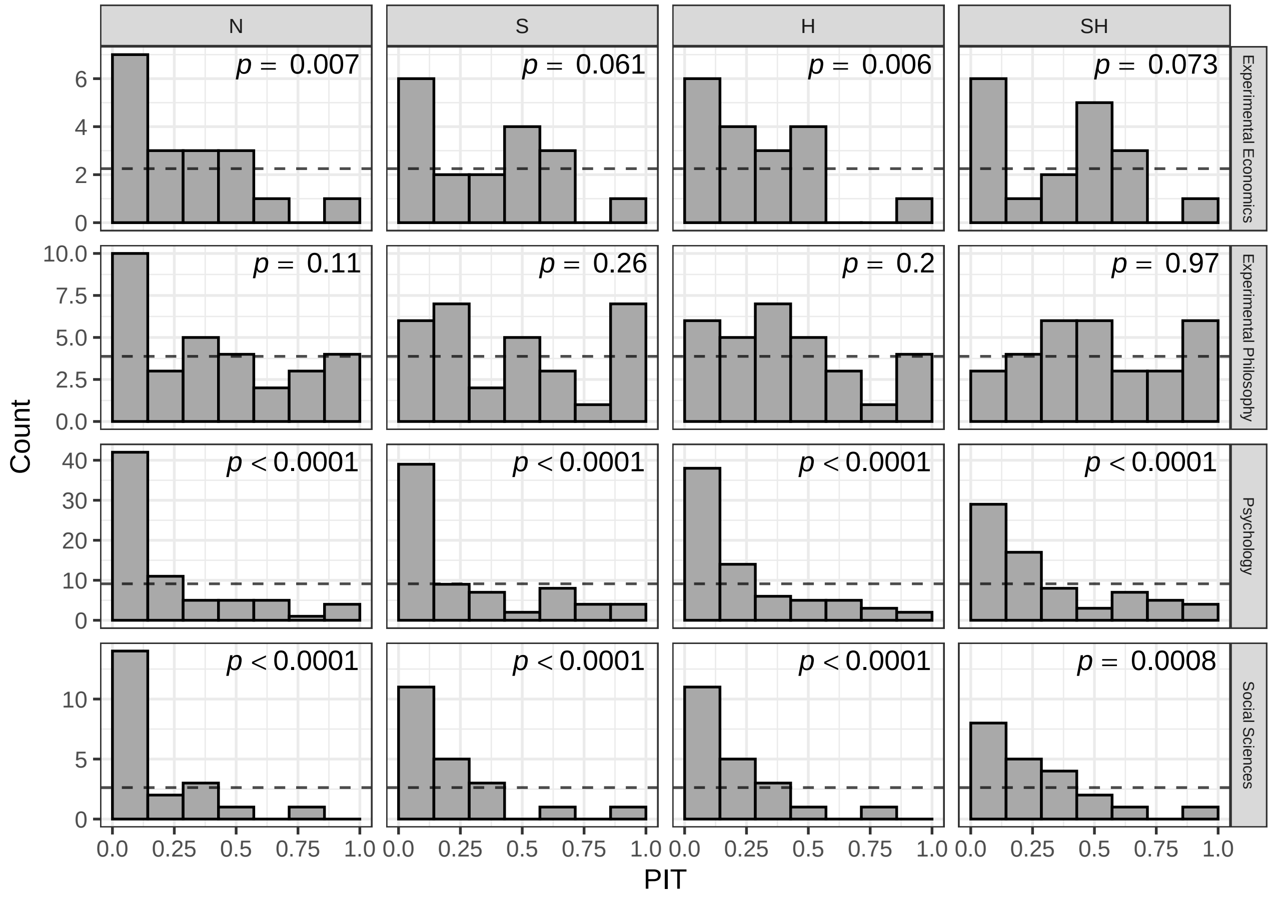

Table 1

```
# Proper scoring rules
# ----------------------------------------------------------------------------------
# Helper functions for proper scoring rules for gaussian predictive distribution
QS <- function(mu, sigma, y) -2/sigma*dnorm(x = (y - mu)/sigma) + 1/(2*sqrt(pi)*sigma)

LS <- function(mu, sigma, y) (y - mu)^2/(2*sigma^2) + log(sigma) + 0.5*log(2*pi)

CRPS <- function(mu, sigma, y) {
  sigma*((y - mu)/sigma*(2*pnorm(q = (y - mu)/sigma) - 1) + 2*dnorm(x = (y - mu)/sigma) - 1/sqrt(pi))
}

gaussian_scores <- list("QS" = QS,
                        "LS" = LS,
                        "CRPS" = CRPS)

get_scores <- function(theta_o, theta_r, sigma_o, sigma_r, tau = tau_default,
                       scoring_rules = gaussian_scores) {
  params <- get_params_all_methods(theta_o = theta_o, sigma_o = sigma_o,
                                   sigma_r = sigma_r, tau = tau)
  scores <- lapply(seq_along(scoring_rules), function(i) {
    score_i <- lapply(params, function(method) {
      scoring_rules[[i]](mu = method$mu, sigma = method$sigma, y = theta_r)
    })
    data.frame(score_i, "Type" = names(scoring_rules)[i])
  })
  scores_df <- do.call(rbind, scores)
  return(scores_df)
}

# Compute mean scores
scores <- with(replication_data, get_scores(theta_o = FZ_OS, theta_r = FZ_RS,
                                            sigma_o = FZ_se_OS, sigma_r = FZ_se_RS))
mean_score_data <- data.frame(scores, replication_data) %>%
  gather(key = "Method", value = "Score", levels_methods) %>%
  group_by(Project, Method, Type) %>%
  summarise(mean_Score = mean(Score),
            SE_mean_Score = sd(Score)/sqrt(n())) %>%
  ungroup()


# Miscalibration tests from Held, Rufibach, Balabdaoui (2010)
# ----------------------------------------------------------------------------------
# Note: LS/DSS scoring rules without constant terms were used in paper,
# whereas the definitions in Gneiting & Katzfuss (2014) involve also constants

# Unconditional miscalibration tests
score_calib_test <- function(theta_o, theta_r, sigma_o, sigma_r, tau = tau_default) {
  # scoring rules according to definitions in paper
  scoring_rules_test <- list("LS" = function(mu, sigma, y)  0.5*(log(sigma^2) + ((y - mu)/sigma)^2),
                             "CRPS" = CRPS)
  scores <- get_scores(theta_o = theta_o, theta_r = theta_r, sigma_o = sigma_o,
                       sigma_r = sigma_r, tau = tau, scoring_rules = scoring_rules_test)
  params <- get_params_all_methods(theta_o = theta_o, sigma_o = sigma_o, sigma_r = sigma_r, tau = tau)
  n <- length(theta_o)

  test_log <- lapply(seq(ncol(scores) - 1), function(i) {
    mean_LS <- mean(scores[scores$Type == "LS",i])
    expectation <- 0.5 + mean(log(params[[i]]$sigma))
```

```r
    variance <- 1/(2*n)
    test_statistic <- (mean_LS - expectation)/sqrt(variance)
    test_pvalue <- 2*pnorm(q = abs(test_statistic), lower.tail = FALSE)
    data.frame("t" = test_statistic, "pvalue" = test_pvalue, "Test" = "LS",
               "Method" = colnames(scores)[i])
  })
  test_crps <- lapply(seq(ncol(scores) - 1), function(i) {
    mean_CRPS <- mean(scores[scores$Type == "CRPS",i])
    expectation <- 1/sqrt(pi)*mean(params[[i]]$sigma)
    variance <- 0.1627516/n^2 * sum(params[[i]]$sigma^2)
    test_statistic <- (mean_CRPS - expectation)/sqrt(variance)
    test_pvalue <- 2*pnorm(q = abs(test_statistic), lower.tail = FALSE)
    data.frame("t" = test_statistic, "pvalue" = test_pvalue, "Test" = "CRPS",
               "Method" = colnames(scores)[i])
  })
  tests_df <- do.call(rbind, c(test_log, test_crps))
  return(tests_df)
}

# Score regression calibration tests
# 1) DSS_i = a + b*log(sigma_i) + e_i
# ===> e_i homoscedastic, H0: a = 0.5, b = 1
# 2) CRPS_i = c + d*sigma_i + e_i
# ===> e_i heteroscedastic (w_i = 1/sigma^2_i), H0: c = 0, d = 1/sqrt(pi)
score_calib_regr_test <- function(theta_o, theta_r, sigma_o, sigma_r, tau = tau_default) {
  # scoring rules according to definitions in paper
  scoring_rules_test <- list("DSS" = function(mu, sigma, y) 0.5*(log(sigma^2) + ((y - mu)/sigma)^2),
                             "CRPS" = CRPS)
  scores <- get_scores(theta_o = theta_o, theta_r = theta_r, sigma_o = sigma_o,
                       sigma_r = sigma_r, tau = tau, scoring_rules = scoring_rules_test)
  params <- get_params_all_methods(theta_o = theta_o, sigma_o = sigma_o,
                                   sigma_r = sigma_r, tau = tau)

  test_dss <- lapply(seq(ncol(scores) - 1), function(i) {
    dss_i <- scores[scores$Type == "DSS",i]
    sigma_i <- params[[i]]$sigma
    fit_dss_i <- lm(dss_i ~ 1 + log(sigma_i))
    ab_diff_i <- matrix(coef(fit_dss_i) - c(0.5, 1))
    statistic <- t(ab_diff_i) %*% solve(vcov(fit_dss_i)) %*% ab_diff_i
    test_pvalue <- pchisq(q = statistic, 2, lower.tail = FALSE)
    data.frame("t" = statistic, "pvalue" = test_pvalue,
               "Test" = "DSS-Regression", "Method" = colnames(scores)[i])
  })
  test_crps <- lapply(seq(ncol(scores) - 1), function(i) {
    crps_i <- scores[scores$Type == "CRPS",i]
    sigma_i <- params[[i]]$sigma
    fit_crps_i <- lm(crps_i ~ 1 + sigma_i, weights = 1/sigma_i^2)
    cd_diff_i <- matrix(coef(fit_crps_i) - c(0, 1/sqrt(pi)))
    statistic <- t(cd_diff_i) %*% solve(vcov(fit_crps_i)) %*% cd_diff_i
    test_pvalue <- pchisq(q = statistic, 2, lower.tail = FALSE)
    data.frame("t" = statistic, "pvalue" = test_pvalue,
               "Test" = "CRPS-Regression", "Method" = colnames(scores)[i])
  })
  tests_df <- do.call(rbind, c(test_dss, test_crps))
  return(tests_df)
}

# Conduct score based miscalibration tests
miscalibtest_df <- lapply(unique(replication_data$Project), function(project) {
  tmp_data <- replication_data[replication_data$Project == project,]
```

```
  test1 <- with(tmp_data, score_calib_test(theta_o = FZ_OS, theta_r = FZ_RS,
                                           sigma_o = FZ_se_OS, sigma_r = FZ_se_RS))
  test2 <- with(tmp_data, score_calib_regr_test(theta_o = FZ_OS, theta_r = FZ_RS,
                                                sigma_o = FZ_se_OS, sigma_r = FZ_se_RS))
  data.frame(rbind(test1, test2), Project = project)
}) %>%
  bind_rows()

# Summarize 4 tests with harmonic mean of p-values
harmonic_mean <- function(x) 1/(mean(1/x))
hmean_pval_df <- rbind(miscalibtest_df) %>%
  group_by(Project, Method) %>%
  summarise(hmean_pval = harmonic_mean(pvalue)) %>%
  ungroup()

# Create table with scores and harmonic mean of p-values
score_table_df <- mean_score_data %>%
  left_join(hmean_pval_df, by = c("Project", "Method")) %>%
  mutate(Project = factor(Project, levels = levels_projects),
         Method = factor(Method, levels = levels_methods),
         Type = factor(Type, levels = levels_scores))

score_table <- tabular(Project * Method ~ ((Type*Heading()*mean_Score)*
                       Format(digits = 1) + formatPval(hmean_pval)*Justify(c,l))*
                       Heading()*identity, data = score_table_df)
colLabels(score_table)[1:1] <- "Score Type"
colLabels(score_table)[2,1:3] <- levels_scores
colLabels(score_table)[2,4] <- "$\\mathring{p}$"
rowLabels(score_table)[2,1] <- "$n = 18$"
rowLabels(score_table)[6,1] <- "$n = 31$"
rowLabels(score_table)[10,1] <- "$n = 73$"
rowLabels(score_table)[14,1] <- "$n = 21$"
toKable(score_table, booktabs = TRUE)
```

		Score Type			
Project	Method	QS	LS	CRPS	$\mathring{p}$
Experimental Economics	N	−0.83	0.34	0.21	0.013
$n = 18$	S	−1.17	0.17	0.17	0.056
	H	−1.14	0.18	0.21	0.24
	SH	−1.32	0.02	0.17	0.79
Experimental Philosophy	N	−1.33	−0.05	0.12	0.0005
$n = 31$	S	−1.46	−0.06	0.12	0.0002
	H	−1.51	−0.18	0.12	0.81
	SH	−1.67	−0.20	0.11	0.66
Psychology	N	−0.07	0.87	0.22	< 0.0001
$n = 73$	S	−0.15	0.86	0.19	< 0.0001
	H	−0.55	0.51	0.22	< 0.0001
	SH	−0.85	0.27	0.18	< 0.0001
Social Sciences	N	−0.17	0.85	0.22	< 0.0001
$n = 21$	S	−0.58	0.54	0.19	< 0.0001
	H	−0.67	0.55	0.21	< 0.0001
	SH	−1.17	0.25	0.18	0.01

Table 2

```
# Helper functions to compute probability for significant replication in same direction
# under gaussian predictive distribution
prob_signif <- function(mu, sigma, sigma_r, alpha = 0.05) {
  direction <- ifelse(mu >= 0, 1, -1)
```

```
  p_significant <- pnorm(direction*qnorm(1 - alpha/2), mean = mu/sigma_r, sd = sigma/sigma_r,
                         lower.tail = ifelse(direction == 1, FALSE, TRUE))
  return(p_significant)
}

get_prob_signif <- function(theta_o, sigma_o, sigma_r, tau = tau_default, alpha = 0.05) {
  params <- get_params_all_methods(theta_o = theta_o, sigma_o = sigma_o, sigma_r = sigma_r, tau = tau)
  p_significant <- lapply(params, function(method) {
    prob_signif(mu = method$mu, sigma = method$sigma, sigma_r = sigma_r, alpha = alpha)
    })
  return(p_significant)
}

# Compute binary predictions under all prediction methods and merge to one data set
binary_pred_stat <- with(replication_data,
                         get_prob_signif(theta_o = FZ_OS, sigma_o = FZ_se_OS,
                                         sigma_r = FZ_se_RS, alpha = 0.05)) %>%
  data.frame(replication_data) %>%
  gather(key = "Method", value = "p", levels_methods)

binary_pred_df <- pm_data %>%
  mutate(p = Market_Belief,
         Method = "PM") %>%
  rbind(binary_pred_stat) %>%
  mutate(Project = factor(Project, levels = levels_projects),
         Method = factor(Method, levels = levels_methods_pm))

# Table: compare expected vs. observed number of significant replications
exp_obs_table_df <- binary_pred_df %>%
  group_by(Project, Method) %>%
  summarise(N = n(),
            Obs = sum(pval_RS < 0.05),
            Exp = sum(p),
            X = (Obs - Exp)^2/Exp + ((N - Obs) - (N - Exp))^2/(N - Exp),
            pvalue = formatPval(pchisq(X, df = 1, lower.tail = FALSE))) %>%
  ungroup()

exp_obs_table <- tabular(Project*Method ~ (Obs + Exp*Format(digits = 3) +
                         pvalue*Justify(l, l))*Heading()*identity*DropEmpty(empty = "."),
                         data = exp_obs_table_df)
colLabels(exp_obs_table)[1,] <- c("Observed", "Expected", "$p\\,$-value")
rowLabels(exp_obs_table)[2,1] <- "$n = 18$"
rowLabels(exp_obs_table)[7,1] <- "$n = 31$"
rowLabels(exp_obs_table)[11,1] <- "$n = 73$"
rowLabels(exp_obs_table)[15,1] <- "$n = 21$"
toKable(exp_obs_table, booktabs = TRUE)
```

Project	Method	Observed	Expected	p-value
Experimental Economics	N	11	15.0	0.012
$n = 18$	S	11	13.6	0.16
	H	11	14.3	0.057
	SH	11	12.4	0.49
	PM	11	13.6	0.16
Experimental Philosophy	N	23	27.8	0.004
$n = 31$	S	23	26.2	0.11
	H	23	26.5	0.076
	SH	23	24.1	0.65
Psychology	N	24	55.4	< 0.0001
$n = 73$	S	24	49.2	< 0.0001
	H	24	53.5	< 0.0001
	SH	24	45.9	< 0.0001
Social Sciences	N	13	19.9	< 0.0001
$n = 21$	S	13	19.2	< 0.0001
	H	13	18.9	< 0.0001
	SH	13	17.6	0.006
	PM	13	13.3	0.89

Table 3

```r
# Table: mean (normalized) Brier score and Spiegelhalter's z-statistic with p-value
brier_table_df <- binary_pred_df %>%
  mutate(y = as.integer(pval_RS < 0.05),
         brier = (y - p)^2) %>%
  group_by(Project, Method) %>%
  summarise(mean_brier0 = mean(y)*(1 - mean(y)),
            mean_brier = mean(brier),
            mean_brier_norm = (mean_brier0 - mean_brier)/mean_brier0,
            z = sum((y - p)*(1 - 2*p))/sqrt(sum((1 - 2*p)^2*p*(1 - p))),
            pvalue = formatPval(2*pnorm(q = abs(z), lower.tail = FALSE)),
            se_mean_brier = sd(brier)/sqrt(n())) %>%
  ungroup()

brier_table <- tabular(Project * Method ~ ((mean_brier + mean_brier_norm)*Format(digit = 1) +
                       (z*Format(digit = 1)*Justify(r) + Justify(l)*pvalue))*Heading()*
                       identity*DropEmpty(empty = "."), data = brier_table_df)
colLabels(brier_table)[1,] <- c("BS", "BS norm", "$z$", "$p\\,$-value")
rowLabels(brier_table)[2,1] <- "$n = 18$"
rowLabels(brier_table)[7,1] <- "$n = 31$"
rowLabels(brier_table)[11,1] <- "$n = 73$"
rowLabels(brier_table)[15,1] <- "$n = 21$"
toKable(brier_table, booktabs = TRUE)
```

Project	Method	BS	BS norm	z	p-value
Experimental Economics	N	0.271	−0.139	2.5	0.013
$n = 18$	S	0.226	0.048	1.1	0.25
	H	0.262	−0.104	2.0	0.042
	SH	0.227	0.046	0.8	0.43
	PM	0.243	−0.021	1.5	0.15
Experimental Philosophy	N	0.193	−0.007	3.3	0.0009
$n = 31$	S	0.173	0.097	2.1	0.039
	H	0.170	0.110	1.6	0.11
	SH	0.148	0.229	0.2	0.83
Psychology	N	0.394	−0.784	10.0	< 0.0001
$n = 73$	S	0.335	−0.518	7.8	< 0.0001
	H	0.363	−0.644	8.5	< 0.0001
	SH	0.289	−0.308	5.2	< 0.0001
Social Sciences	N	0.346	−0.468	7.2	< 0.0001
$n = 21$	S	0.324	−0.374	5.5	< 0.0001
	H	0.310	−0.316	4.9	< 0.0001
	SH	0.272	−0.155	3.4	0.0006
	PM	0.114	0.519	− 2.0	0.044

Figure 9

```r
# Obtain calibaration slope by logistic regression
apply_grid <- expand.grid(project = levels_projects,
                          method = levels_methods_pm)

calib_slope_df <- apply(apply_grid, 1, function(par) {
  tmp_data <- binary_pred_df[binary_pred_df$Project == par["project"] &
                             binary_pred_df$Method == par["method"],]
  tmp_data$y <- as.integer(tmp_data$pval_RS < 0.05)
  tmp_data$logit_p <- qlogis(tmp_data$p)
  logist_fit <- try(glm(y ~ logit_p, family = "binomial", data = tmp_data))
  if(inherits(logist_fit, "try-error")) {
    NA_df <- data.frame("CI_lower" = NA, "Slope" = NA, "CI_upper" = NA,
                        "Method" = par["method"], "Project" = par["project"])
    return(NA_df)
  } else {
    slope_ci <- confint.default(logist_fit)
    data.frame("CI_lower" = slope_ci[2,1], "Slope" = unname(coef(logist_fit)[2]),
               "CI_upper" = slope_ci[2,2], "Method" = par["method"], "Project" = par["project"])
  }
}) %>%
  bind_rows() %>%
  mutate(Method = factor(Method, levels = rev(levels_methods_pm)),
         Project = factor(Project, levels = rev(levels_projects),
                          labels = rev(labels_projects2lines)))

## Error in family$linkfun(mustart) :
##   Argument mu must be a nonempty numeric vector
## Error in family$linkfun(mustart) :
##   Argument mu must be a nonempty numeric vector

calibslope_plot <- ggplot(data = filter(calib_slope_df, Slope < 100),
                          aes(x = Project, y = Slope, color = Method)) +
  geom_hline(yintercept = 1, lty = 3, alpha = 0.8) +
  geom_pointrange(aes(ymin = CI_lower, ymax = CI_upper), size = 0.5, fatten = 3,
                  position = position_dodge2(width = 0.7)) +
  labs(y = "Calibration slope") +
  guides(color = guide_legend(reverse = TRUE)) +
```

```
  scale_color_manual(name = "Method", values = colors_methods) +
  coord_flip() +
  theme_bw()

# Compute AUC
auc_df <- binary_pred_df %>%
  group_by(Project, Method) %>%
  mutate(y = as.integer(pval_RS < 0.05)) %>%
  summarise(CI_lower = confIntAUC(cases = p[y == 1], controls = p[y == 0])$lower[2],
            AUC = confIntAUC(cases = p[y == 1], controls = p[y == 0])$AUC[2],
            CI_upper = confIntAUC(cases = p[y == 1], controls = p[y == 0])$upper[2]) %>%
  ungroup() %>%
  mutate(Method = factor(Method, levels = rev(levels_methods_pm)),
         # Perfect separation for PM in social sciences data set -> set CI-limits to AUC
         CI_lower = ifelse(Method == "PM" & Project == "Social Sciences", AUC, CI_lower),
         CI_upper = ifelse(Method == "PM" & Project == "Social Sciences", AUC, CI_upper),
         Project = factor(Project, levels = rev(levels_projects),
                          labels = rev(labels_projects2lines)))

auc_plot <- ggplot(data = auc_df, aes(x = Project, y = AUC, color = Method)) +
  geom_hline(yintercept = 0.5, lty = 3, alpha = 0.8) +
  geom_pointrange(aes(ymin = CI_lower, ymax = CI_upper), size = 0.5,
                  position = position_dodge2(width = 0.7), fatten = 3) +
  coord_flip() +
  guides(color = guide_legend(reverse = TRUE)) +
  labs(x = NULL) +
  scale_y_continuous(breaks = seq(0.25, 1, 0.25)) +
  scale_color_manual(name = "Method", values = colors_methods) +
  theme_bw()

# Combine calibration slope and AUC into one plot
ggarrange(calibslope_plot, auc_plot, ncol = 2, common.legend = TRUE, legend = "right",
          labels = list("(A)", "(B)"), font.label = list(face = "plain"))
```

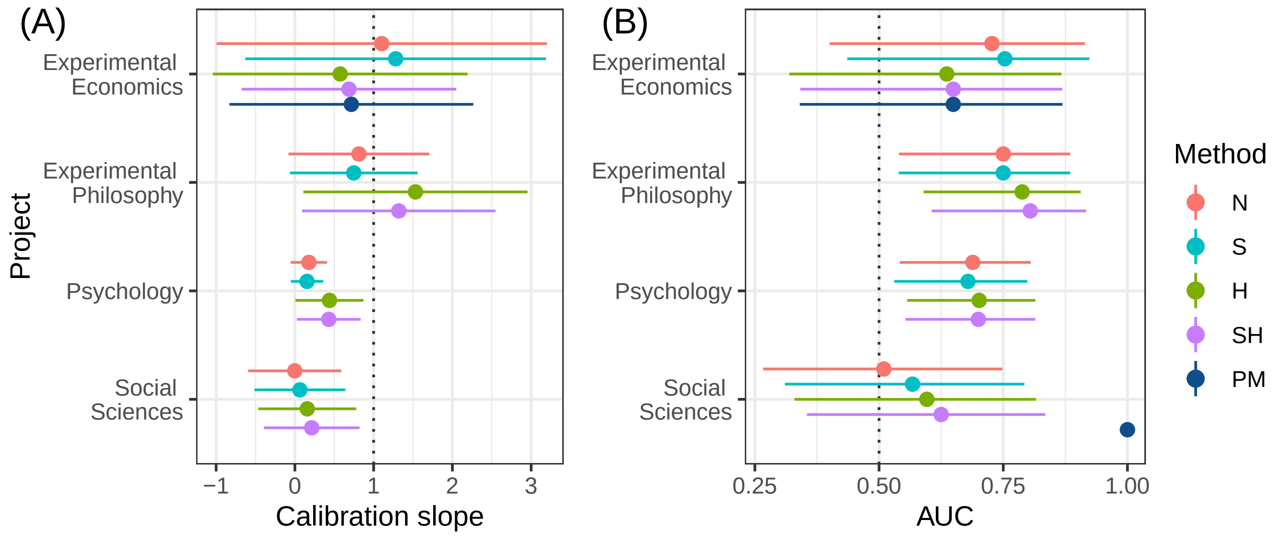

Figure 10

```
# Compute mean scores for different values of tau
tau_seq <- seq(0, 0.4, 0.001)
models <- list("H" = prediction_methods[["H"]],
               "SH" = prediction_methods[["SH"]])
```

```
apply_grid <- expand.grid(project = unique(replication_data$Project),
                          score_type = names(gaussian_scores),
                          model = names(models))

sensitivity_results <- apply(apply_grid, 1, function(par) {
  tmp_data <- replication_data[replication_data$Project == par["project"],]
  score_function <- function(tau) {
    params <- with(tmp_data, models[[par["model"]]](theta_o = FZ_OS, sigma_o = FZ_se_OS,
                                                     sigma_r = FZ_se_RS, tau = tau))
    scores <- gaussian_scores[[par["score_type"]]](mu = params$mu, sigma = params$sigma,
                                                    y = tmp_data$FZ_RS)
    return(mean(scores))
  }
  optim_result <- optim(par = 0.001, fn = score_function, method = "L-BFGS-B",
                        lower = 0, upper = 10)
  optim_df <- data.frame(tau_hat = optim_result$par,
                         mean_score = optim_result$value,
                         Score_Type = par["score_type"],
                         Method = par["model"],
                         Project = par["project"])
  scores_df <- data.frame(tau = tau_seq,
                          mean_score = sapply(tau_seq, score_function),
                          Score_Type = par["score_type"],
                          Method = par["model"],
                          Project = par["project"])
  return(list(scores = scores_df, optim = optim_df))
})

score_df <- do.call(rbind, lapply(sensitivity_results, function(list) list$scores)) %>%
  mutate(Method = factor(Method, levels = levels_methods),
         Project = factor(Project, levels = levels_projects),
         Score_Type = factor(Score_Type, levels = levels_scores))
optim_df <- do.call(rbind, lapply(sensitivity_results, function(list) list$optim)) %>%
  mutate(Method = factor(Method, levels = levels_methods),
         Project = factor(Project, levels = levels_projects),
         Score_Type = factor(Score_Type, levels = levels_scores)) %>%
  group_by(Score_Type) %>%
  mutate(padding_score = sd(mean_score)*0.5) %>%
  ungroup()

# Plot
score_df %>%
  ggplot(aes(x = tau, y = mean_score, color = Method)) +
  geom_vline(xintercept = 0.08, lty = 2, alpha = 0.8) +
  geom_line(size = 0.8, alpha = 0.9) +
  geom_point(data = optim_df, size = 2, shape = 4, aes(x = tau_hat, y = mean_score, color = Method)) +
  geom_text(data = filter(optim_df, Method == "H"), show.legend = FALSE,
            aes(x = tau_hat, y = mean_score + padding_score, label = round(tau_hat, 2))) +
  geom_text(data = filter(optim_df, Method == "SH"), show.legend = FALSE,
            aes(x = tau_hat, y = mean_score - padding_score, label = round(tau_hat, 2))) +
  facet_grid(Score_Type ~ Project, scales = "free_y") +
  labs(x = expression(tau), y = "Mean score") +
  scale_color_manual(name = "Method", values = colors_methods) +
  theme_bw() +
  theme(legend.position = "bottom")
```

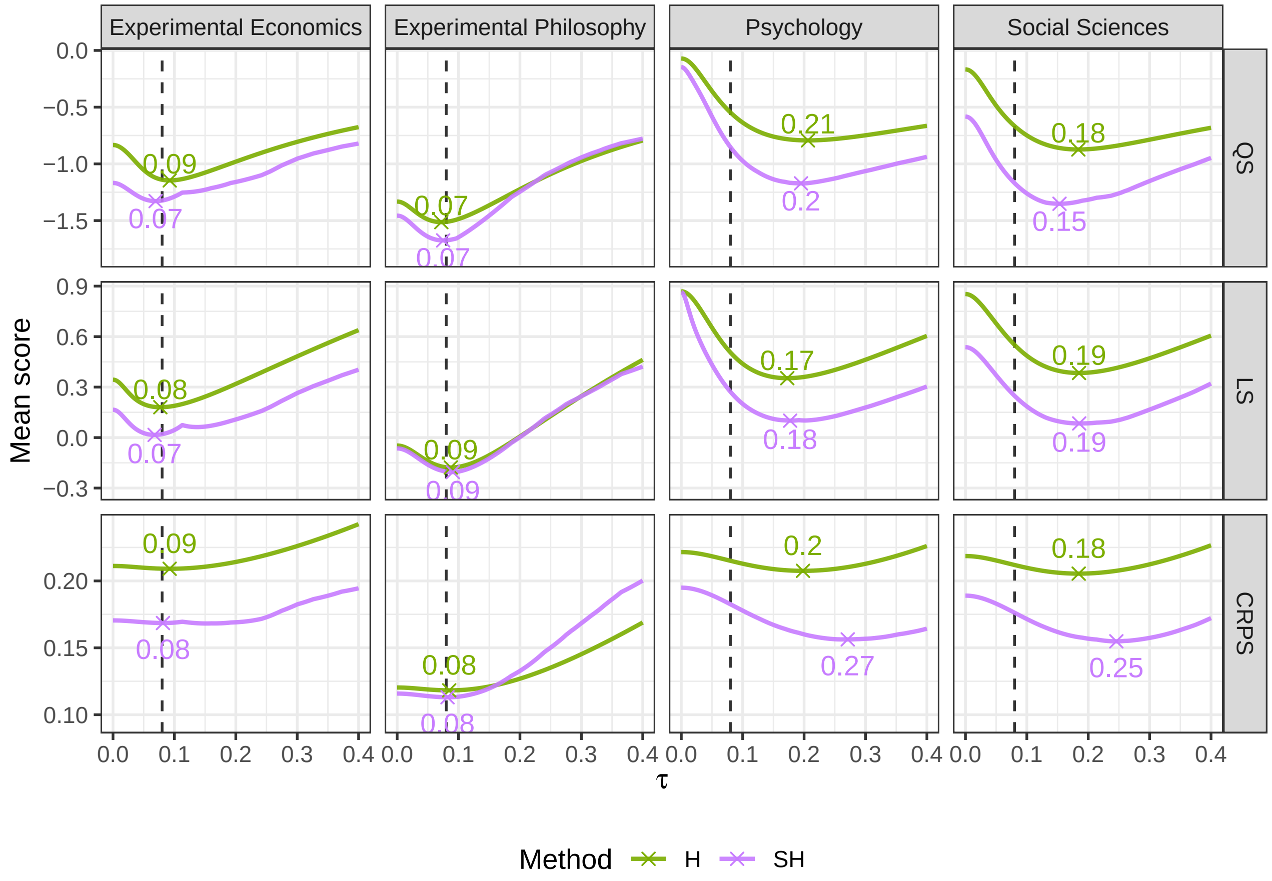

Evaluations from supplement B

Figure 1

```r
# Helper function to compute required relative sample size c
sampleSizeReplication <- function(t_o, d = 0, power = 0.8, level = 0.05,
                                  alternative = "two.sided", prior = "flat") {

  # specify direction and critical value
  direction <- ifelse(t_o >= 0, 1, -1)
  alt <- ifelse(alternative == "two.sided", 2, 1)
  critical_value <- direction*qnorm(1 - level/alt)

  # define power function depending on prior
  if (prior == "flat") s <- 1
  if (prior == "sceptical") s <- pmax(1 - (1 + d)/t_o^2, 0)
  powerFun <- function(c) {
    power <- pnorm(q = critical_value,
                   mean = s*t_o*sqrt(c),
                   sd = sqrt(s*(c + d*c) + 1 + d*c),
                   lower.tail = ifelse(direction == 1, FALSE, TRUE))
    return(power)
  }

  # find required relative sample size (upper limit c = 100)
  c <- try(uniroot(f = function(c) powerFun(c) - power, lower = 0, upper = 100)$root)
  if (class(c) == "try-error") return(NA)
  return(c)
}
```

```
apply_grid <- expand.grid(t_o = seq(0, 4.5, 0.01),
                         d = seq(0, 2, 0.4),
                         prior = c("flat", "sceptical"))
samplesize_df <- apply(apply_grid, 1, function(par) {
  c <- sampleSizeReplication(t_o = as.double(par[1]), d = as.double(par[2]), prior = par[3])
  result <- data.frame(c, t_o = as.double(par[1]), d = as.double(par[2]), Prior = par[3])
  return(result)
}) %>%
  bind_rows() %>%
  mutate(Prior = case_when(Prior == "flat" ~ "Heterogeneity",
                           Prior == "sceptical" ~ "Shrinkage + Heterogeneity"))

## Plot of c needed to achieve 80% power
ggplot(data = samplesize_df, aes(x = t_o, y = c, color = d)) +
  geom_hline(yintercept = 1, lty = 2) +
  geom_line(aes(group = factor(d)), size = 0.8) +
  facet_wrap(~ Prior, ncol = 1) +
  labs(x = bquote(italic(t)[o]), y = bquote(italic(c))) +
  guides(color = guide_colorbar(title = bquote(italic(~~d)))) +
  scale_color_viridis_c() +
  scale_y_log10(breaks = c(0.5, 1, 2, 10, 100)) +
  scale_x_continuous(sec.axis = sec_axis(trans = ~z_to_p(.),
                                         breaks = z_to_p(seq(0, 4, 1)),
                                         labels = pval_labels,
                                         name = expression(italic(p)[o])),
                     breaks = seq(0, 5, 1))  +
  theme_bw()
```

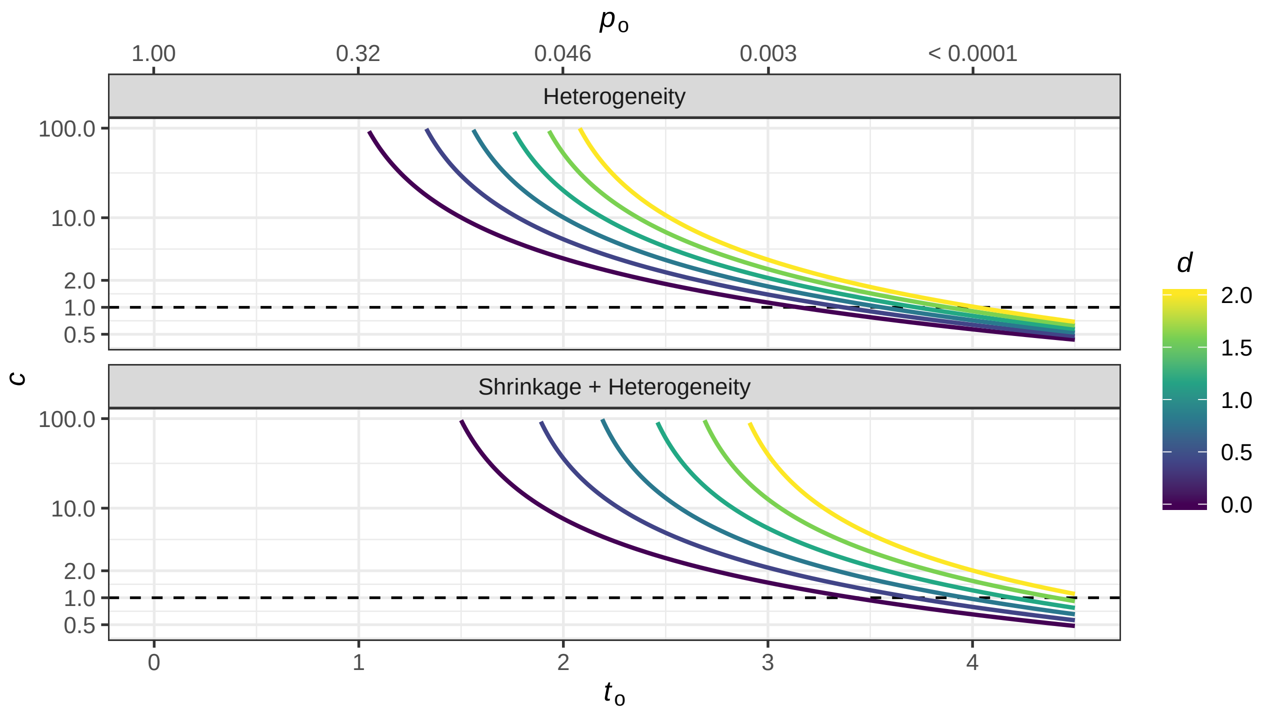

Table 1

```
# Conduct kolmogorov-smirnov test for pit values to test whether U(0, 1)
PIT_ks_table <- PIT_ks %>%
  mutate(Project = factor(Project, levels = levels_projects))

pit_ks_table <- tabular(Project * Method ~ (t*Format(digits = 1) + formatPval(pvalue)*
                        Justify(l,l))*Heading()*identity, data = PIT_ks_table)
```

```r
colLabels(pit_ks_table)[1,] <- c("Test statistic", "$p\\,$-value")
rowLabels(pit_ks_table)[2,1] <- "$n = 18$"
rowLabels(pit_ks_table)[6,1] <- "$n = 31$"
rowLabels(pit_ks_table)[10,1] <- "$n = 73$"
rowLabels(pit_ks_table)[14,1] <- "$n = 21$"
toKable(pit_ks_table, booktabs = TRUE)
```

Project	Method	Test statistic	p-value
Experimental Economics	N	0.38	0.007
$n = 18$	S	0.30	0.061
	H	0.39	0.006
	SH	0.29	0.073
Experimental Philosophy	N	0.21	0.11
$n = 31$	S	0.18	0.26
	H	0.19	0.20
	SH	0.08	0.97
Psychology	N	0.48	< 0.0001
$n = 73$	S	0.41	< 0.0001
	H	0.44	< 0.0001
	SH	0.36	< 0.0001
Social Sciences	N	0.61	< 0.0001
$n = 21$	S	0.52	< 0.0001
	H	0.54	< 0.0001
	SH	0.42	0.0008

Figure 2

```r
# Determine shrinkage factor s under S and SH model
shrinkage_factor <- function(t_o, d) pmax(1 - (1 + d)/t_o^2, 0)
shrinkage_df <- replication_data %>%
  mutate(S = shrinkage_factor(t_o = FZ_OS/FZ_se_OS, d = 0),
         SH = shrinkage_factor(t_o = FZ_OS/FZ_se_OS, d = 0.08^2/FZ_se_OS^2),
         Project = factor(Project, levels = levels_projects,
                          labels = labels_projects2lines)) %>%
  gather(key = "Method", value = "s", S, SH)

ggplot(data = shrinkage_df, aes(x = Project, y = s)) +
  geom_boxplot(alpha = 0.5, fill = "lightgrey") +
  geom_quasirandom(size = 1.5, shape = 21, alpha = 0.7, fill = "grey20") +
  facet_wrap(~ Method) +
  labs(y = expression(italic(s))) +
  theme_bw() +
  theme(axis.text.x = element_text(angle = 90))
```

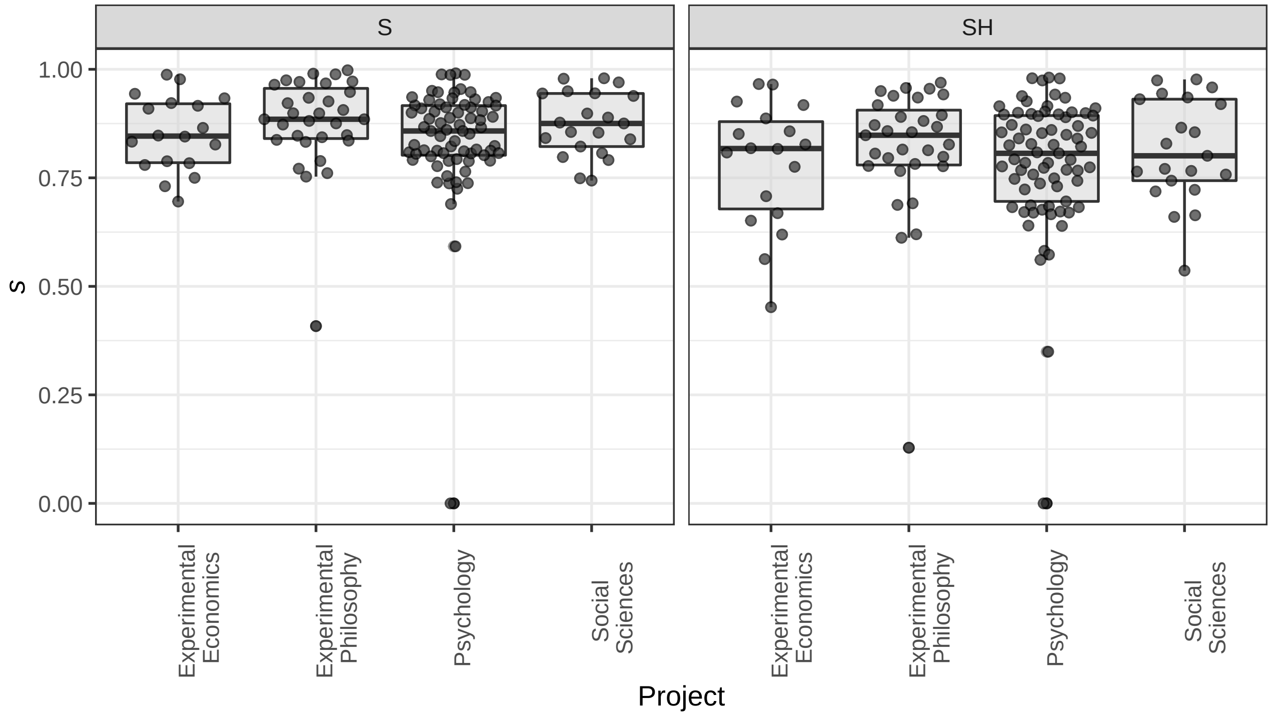

Figure 3

```
# Plot of mean scores with standard errors
mean_score_data_plot <- mean_score_data %>%
  # same order as in tables
  mutate(Method = factor(Method, levels = rev(levels_methods)),
         Project = factor(Project, levels = rev(levels_projects),
                          labels = rev(labels_projects2lines)))

ggplot(data = mean_score_data_plot, aes(x = Project, y = mean_Score, color = Method)) +
  geom_pointrange(aes(ymin = mean_Score - SE_mean_Score, y = mean_Score,
                      ymax = mean_Score + SE_mean_Score),
                  position = position_dodge2(width = 0.5), fatten = 3) +
  facet_grid(~ Type, scales = "free") +
  coord_flip() +
  guides(color = guide_legend(reverse = TRUE)) +
  labs(y = expression(paste("Mean score (", ''%+-%'', " se)"))) +
  scale_color_manual(name = "Method", values = colors_methods) +
  theme_bw() +
  theme(legend.position = "bottom")
```

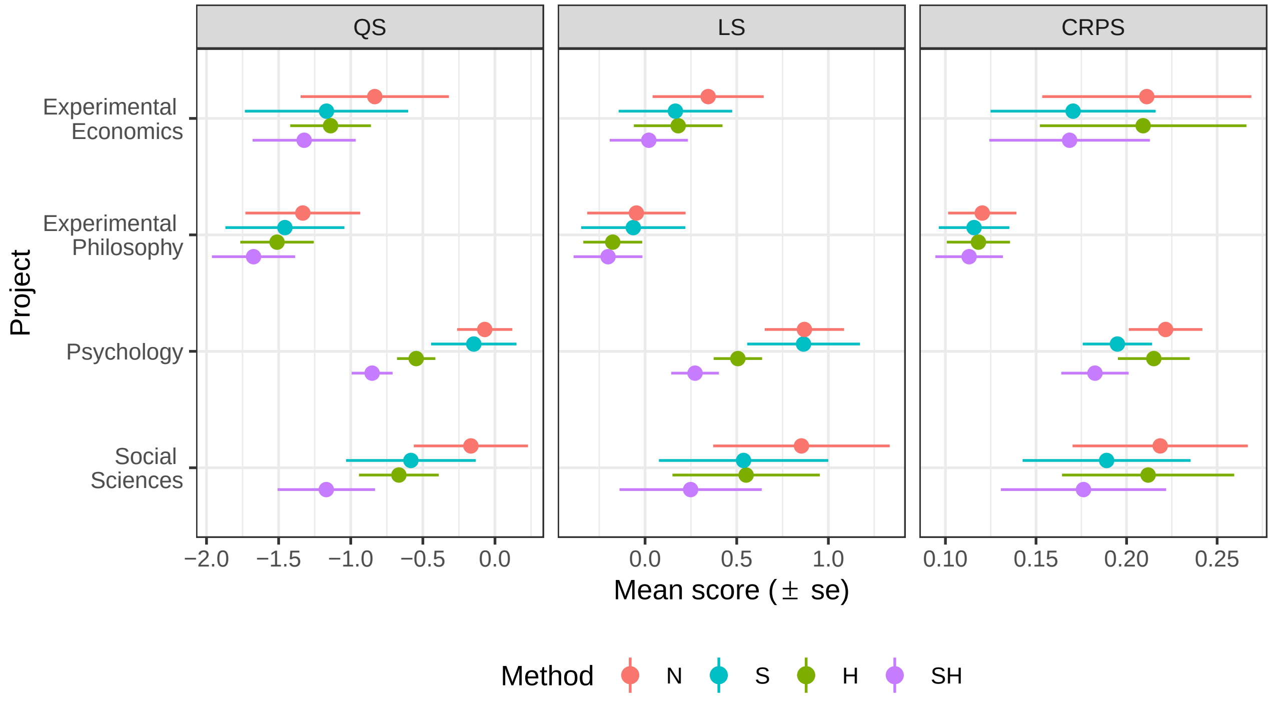

Table 2

```
# Conduct paired tests whether scores different between methods
scores_df <- data.frame(scores, replication_data) %>%
  gather(key = "Method", value = "Score", levels_methods)
apply_grid <- expand.grid(project = unique(scores_df$Project),
                         score_type = names(gaussian_scores))

paired_tests <- apply(apply_grid, 1, function(par) {
  tmp_data <- scores_df[scores_df$Project == par["project"] &
                         scores_df$Type == par["score_type"],]
  test <- pairwise.wilcox.test(x = tmp_data$Score, g = tmp_data$Method,
                              paired = TRUE, p.adjust.method = "none")$p.value
  results <- data.frame("Test" = levels_methods[c(3, 1, 2)],
                        "pvalue" = test[3,],
                        "Project" = par["project"],
                        "Type" = par["score_type"])
}) %>%
  bind_rows() %>%
  mutate(Test = factor(Test, levels = levels_methods[1:3]),
         Project = factor(Project, levels = levels_projects),
         Type = factor(Type, levels_scores),
         pvalue = formatPval(pvalue))


paired_test_table <- tabular(Project*Test ~ (Type * pvalue*Justify(c,l))*
                              Heading()*identity, data = paired_tests)
colLabels(paired_test_table)[3,] <- "$p\\,$-value"
rowLabels(paired_test_table)[2,1] <- "$n = 18$"
rowLabels(paired_test_table)[5,1] <- "$n = 31$"
rowLabels(paired_test_table)[8,1] <- "$n = 73$"
rowLabels(paired_test_table)[11,1] <- "$n = 21$"
toKable(paired_test_table, booktabs = TRUE)
```

		Type		
		QS	LS	CRPS
Project	Test	p-value	p-value	p-value
Experimental Economics	N	0.038	0.016	0.007
$n = 18$	S	0.73	0.77	0.70
	H	0.014	0.005	0.003
Experimental Philosophy	N	0.44	0.95	0.66
$n = 31$	S	0.36	0.79	0.95
	H	0.26	0.28	0.47
Psychology	N	< 0.0001	< 0.0001	< 0.0001
$n = 73$	S	< 0.0001	< 0.0001	< 0.0001
	H	< 0.0001	< 0.0001	< 0.0001
Social Sciences	N	0.001	0.0007	0.0004
$n = 21$	S	0.018	0.07	0.001
	H	0.0003	< 0.0001	0.0005

Table 3

```r
# Score-based miscalibration tests
miscalibtest_table_df <- miscalibtest_df %>%
  mutate(Project = factor(Project, levels = levels_projects))

miscalib_table <- tabular(Project*Method ~ Test*(t*Format(digit = 1) + formatPval(pvalue)*
                          Justify(c,l))*Heading()*identity, data = miscalibtest_table_df)
colLabels(miscalib_table)[1,1] <- "Test type"
colLabels(miscalib_table)[3,] <- rep(c("Statistic", "$p\\,$-value"), 4)
rowLabels(miscalib_table)[2,1] <- "$n = 18$"
rowLabels(miscalib_table)[6,1] <- "$n = 31$"
rowLabels(miscalib_table)[10,1] <- "$n = 73$"
rowLabels(miscalib_table)[14,1] <- "$n = 21$"
toKable(miscalib_table, booktabs = TRUE)
```

		Test type							
		LS		CRPS		DSS-Regression		CRPS-Regression	
Project	Method	Statistic	p-value	Statistic	p-value	Statistic	p-value	Statistic	p-value
Experimental Economics	N	2.85	0.004	2.30	0.021	3.44	0.18	6.01	0.05
$n = 18$	S	2.11	0.035	1.23	0.22	3.40	0.18	7.01	0.03
	H	0.72	0.47	1.52	0.13	2.97	0.23	1.91	0.39
	SH	0.20	0.84	0.57	0.57	0.13	0.94	0.04	0.98
Experimental Philosophy	N	3.84	0.0001	2.02	0.044	3.67	0.16	3.98	0.14
$n = 31$	S	4.09	< 0.0001	2.13	0.033	3.55	0.17	3.79	0.15
	H	0.48	0.63	0.19	0.85	0.19	0.91	0.13	0.94
	SH	0.80	0.42	0.32	0.75	0.33	0.85	0.36	0.83
Psychology	N	12.86	< 0.0001	8.09	< 0.0001	31.13	< 0.0001	44.76	< 0.0001
$n = 73$	S	13.57	< 0.0001	6.68	< 0.0001	45.17	< 0.0001	80.35	< 0.0001
	H	6.25	< 0.0001	5.49	< 0.0001	16.48	0.0003	19.82	< 0.0001
	SH	4.28	< 0.0001	3.86	0.0001	8.18	0.017	9.56	0.008
Social Sciences	N	7.68	< 0.0001	6.02	< 0.0001	6.26	0.044	9.39	0.009
$n = 21$	S	6.00	< 0.0001	4.86	< 0.0001	4.49	0.11	6.14	0.046
	H	4.30	< 0.0001	4.03	< 0.0001	3.81	0.15	5.11	0.078
	SH	2.79	0.005	2.80	0.005	2.88	0.24	3.33	0.19

Figure 4

```
# Plot probabilitity of significant estimate in same direction under different methods
ggplot(binary_pred_df, aes(x = as.numeric(interaction(Method, pval_RS_significant)), y = p)) +
  geom_line(aes(group = Study), alpha = 0.5) +
  geom_point(aes(fill = Method), shape = 21, size = 2, alpha = 0.8) +
  facet_wrap(~ Project) +
  scale_x_continuous(breaks = c(3, 8), labels = unique(binary_pred_df$pval_RS_significant)) +
  scale_fill_manual(name = "Method", values = colors_methods) +
  labs(x = "Statistical significance of replication study",
       y = "Estimated probability of significance") +
  theme_bw() +
  theme(panel.grid.major.x = element_blank())
```

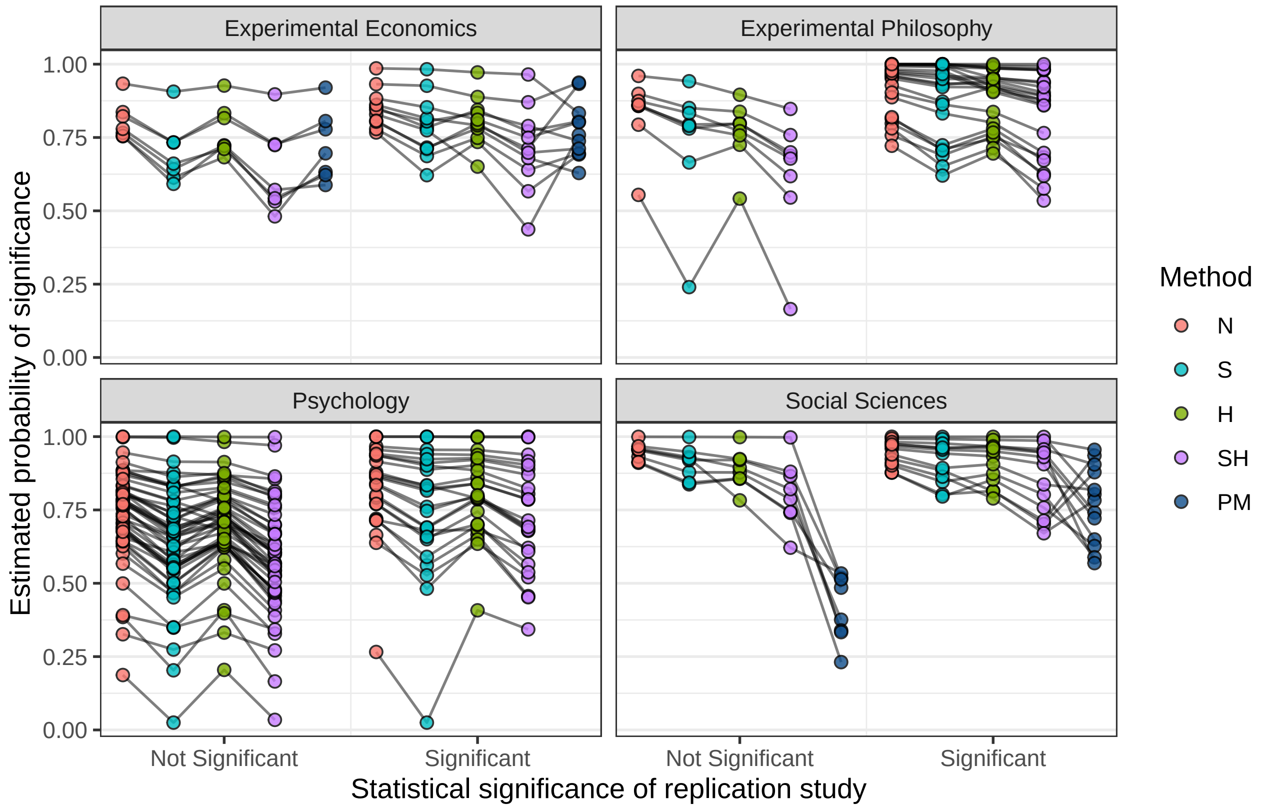

Figure 5

```
# Expected vs. observed #significant replications for different alpha values
apply_grid <- expand.grid(alpha = seq(0.001, 0.06, 0.001),
                          project = unique(replication_data$Project))
exp_obs_df <- apply(apply_grid, 1, function(par) {
  tmp_data <- replication_data[replication_data$Project == par["project"],]
  p <- with(tmp_data, get_prob_signif(theta_o = FZ_OS, sigma_o = FZ_se_OS,
                                      sigma_r = FZ_se_RS, alpha = as.double(par["alpha"])))
  Exp <- sapply(p, sum)
  N <- nrow(tmp_data)
  Obs <- sum(tmp_data$pval_RS < as.double(par["alpha"]))
  X <- (Obs - Exp)^2/Exp + ((N - Obs) - (N - Exp))^2/(N - Exp)
  result <- data.frame(N,
                       Observed = Obs,
                       Expected = Exp,
                       pvalue = formatPval(pchisq(X, df = 1, lower.tail = FALSE)),
                       Method = names(Exp), Project = par["project"],
```

```r
                        alpha = as.double(par["alpha"]))
  return(result)
}) %>%
  bind_rows() %>%
  mutate(Method = factor(Method, levels = levels_methods),
         Project = factor(Project, levels = levels_projects))

ggplot(data = exp_obs_df, aes(x = alpha, y = Observed)) +
  geom_step(aes(color = "Observed")) +
  geom_step(aes(x = alpha, y = Expected, color = Method)) +
  facet_wrap(~ Project, scales = "free") +
  scale_x_log10(breaks = c(0.001, 0.002, 0.005, 0.015, 0.05, 0.15, 0.4, 1)) +
  expand_limits(y = 0) +
  labs(x = expression(alpha), y = "Number of significant replications") +
  scale_color_manual(values = c(Observed = 1, colors_methods)) +
  guides(color = guide_legend(title = "")) +
  theme_bw() +
  theme(legend.position = "bottom")
```

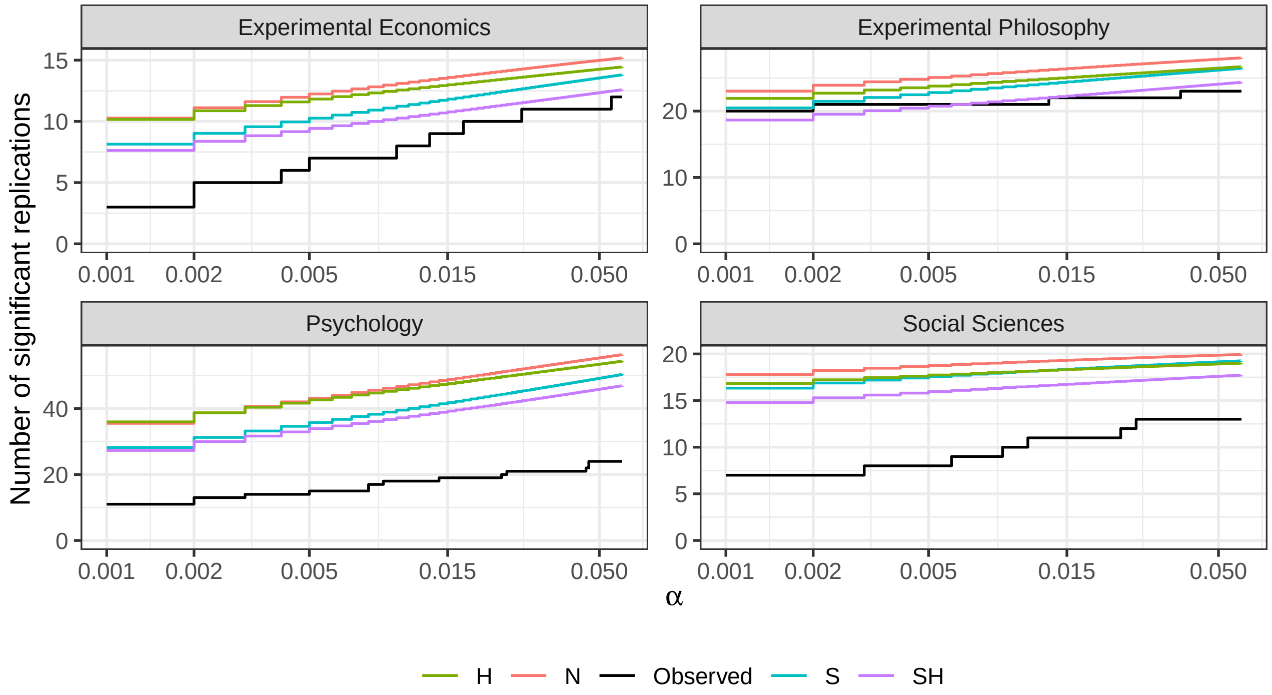

Figure 6

```r
# Plot of mean brier score with standard errors
brier_plot_df <- brier_table_df %>%
  # same order as in tables
  mutate(Method = factor(Method, levels = rev(levels_methods_pm)),
         Project = factor(Project, levels = rev(levels_projects)))

ggplot(data = brier_plot_df, aes(x = Project, y = mean_brier, color = Method)) +
  geom_hline(yintercept = 0.25, lty = 3) +
  geom_pointrange(aes(ymin = mean_brier - se_mean_brier, ymax = mean_brier + se_mean_brier),
                  position = position_dodge2(width = 0.5), fatten = 3) +
  labs(y = bquote(paste("Mean Brier score (", ''%+-%'', " se)"))) +
  guides(color = guide_legend(reverse = TRUE)) +
  expand_limits(y = 0) +
  coord_flip() +
```

```
  scale_color_manual(name = "Method", values = colors_methods) +
  theme_bw()
```

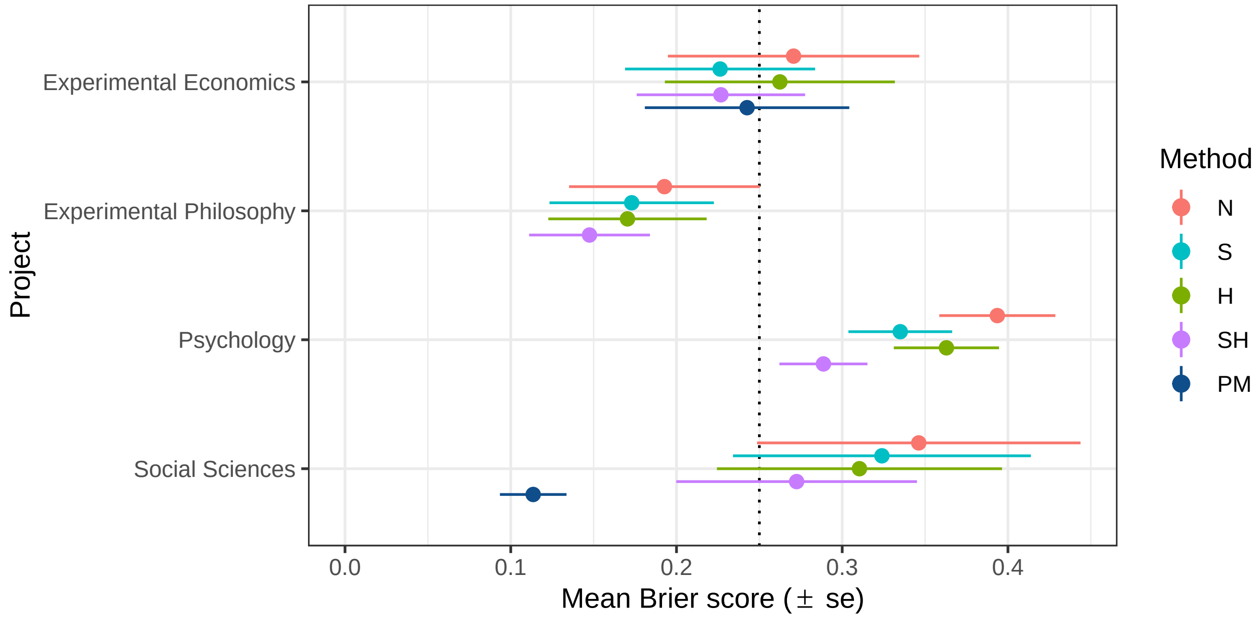

Figure 7

```
# Plot brier score for different levels of alpha
apply_grid <- expand.grid(alpha = seq(0.001, 0.06, 0.001),
                          project = unique(replication_data$Project))
brier_alpha_df <- apply(apply_grid, 1, function(par) {
  tmp_data <- replication_data[replication_data$Project == par["project"],]
  p <- with(tmp_data, get_prob_signif(theta_o = FZ_OS, sigma_o = FZ_se_OS,
                                      sigma_r = FZ_se_RS, alpha = as.double(par["alpha"])))
  y <- as.integer(tmp_data$pval_RS < as.double(par["alpha"]))
  mean_brier <- sapply(p, function(p) mean((y - p)^2))
  result <- data.frame(mean_brier,
                       Method = names(mean_brier),
                       Project = par["project"],
                       alpha = as.double(par["alpha"]))
  return(result)
}) %>%
  bind_rows() %>%
  mutate(Method = factor(Method, levels = levels_methods),
         Project = factor(Project, levels = levels_projects))

ggplot(brier_alpha_df, aes(x = alpha, y = mean_brier, color = Method)) +
  geom_hline(yintercept = 0.25, lty = 3, alpha = 0.8) +
  geom_step() +
  facet_wrap(~ Project) +
  ylim(0, 0.52) +
  scale_x_log10(breaks = c(0.001, 0.002, 0.005, 0.015, 0.05, 0.15, 0.4, 1)) +
  labs(x = bquote(alpha), y = "Mean Brier score") +
  theme_bw() +
  theme(legend.position = "bottom")
```

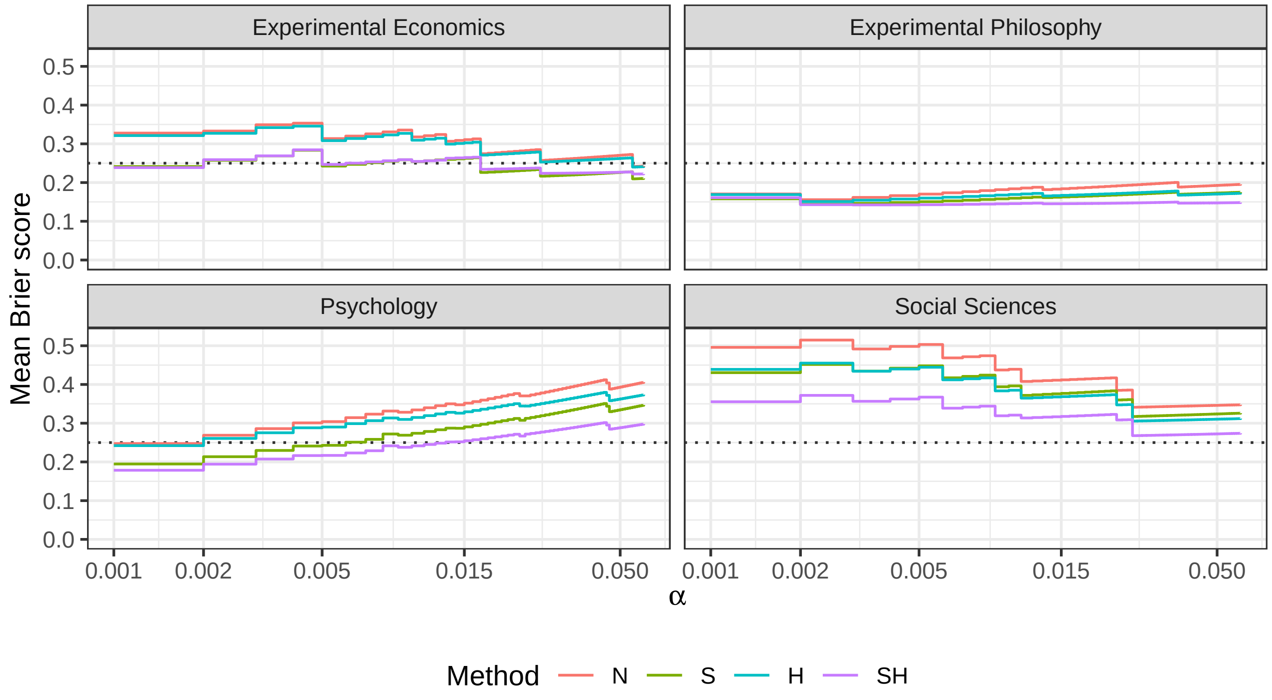

Figure 8

```
# Plot of calibration slope and CI for different alpha
apply_grid <- expand.grid(alpha = seq(0.001, 0.06, 0.001),
                          project = unique(replication_data$Project))
calibslope_alpha_df <- apply(apply_grid, 1, function(par) {
  tmp_data <- replication_data[replication_data$Project == par["project"],]
  p <- with(tmp_data, get_prob_signif(theta_o = FZ_OS, sigma_o = FZ_se_OS,
                                      sigma_r = FZ_se_RS, alpha = as.double(par["alpha"])))
  y <- as.integer(tmp_data$pval_RS < as.double(par["alpha"]))
  results_list <- lapply(names(p), function(method) {
    logist_fit <- try(glm(y ~ qlogis(p[[method]]), family = "binomial"))
    if(inherits(logist_fit, "try-error")) {
      NA_df <- data.frame(CI_lower = NA,
                          Slope = NA,
                          CI_upper = NA,
                          Method = method,
                          Project = par["project"],
                          alpha = as.double(par["alpha"]))
      return(NA_df)
    } else {
      slope_ci <- confint.default(logist_fit)
      data.frame(CI_lower = slope_ci[2,1],
                 Slope = unname(coef(logist_fit)[2]),
                 CI_upper = slope_ci[2,2],
                 Method = method,
                 Project = par["project"],
                 alpha = as.double(par["alpha"]))
    }
  })
  results_df <- bind_rows(results_list)
  return(results_df)
}) %>%
  bind_rows() %>%
  # same order as in tables
  mutate(Method = factor(Method, levels = levels_methods),
```

```
        Project = factor(Project, levels = levels_projects))

ggplot(data = calibslope_alpha_df, aes(x = alpha, y = Slope, color = Method)) +
  geom_hline(yintercept = 1, lty = 2, alpha = 0.8) +
  geom_ribbon(aes(ymin = CI_lower, ymax = CI_upper, fill = Method), alpha = 0.1, lty = 3) +
  geom_line(size = 0.6, alpha = 0.8) +
  facet_wrap(~ Project, scales = "free") +
  scale_x_log10(breaks = c(0.001, 0.002, 0.005, 0.015, 0.05)) +
  labs(x = expression(alpha), y = "Calibration slope") +
  theme_bw() +
  theme(legend.position = "bottom")
```

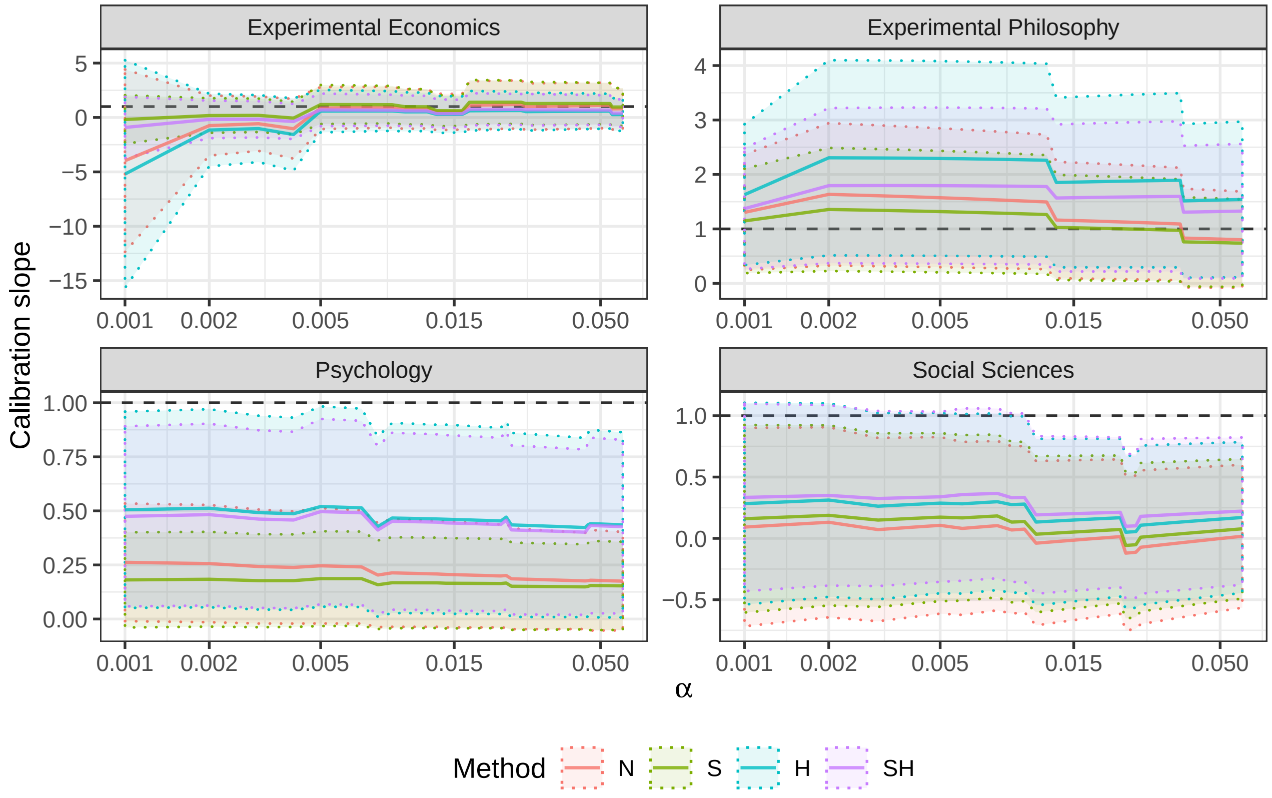

Figure 9

```
# Plot AUC with CI for different alpha values
apply_grid <- expand.grid(alpha = seq(0.001, 0.06, 0.001),
                          project = unique(replication_data$Project))
auc_alpha_df <- apply(apply_grid, 1, function(par) {
  tmp_data <- replication_data[replication_data$Project == par["project"],]
  p <- with(tmp_data, get_prob_signif(theta_o = FZ_OS, sigma_o = FZ_se_OS,
                                       sigma_r = FZ_se_RS, alpha = as.double(par["alpha"])))
  y <- as.integer(tmp_data$pval_RS < as.double(par["alpha"]))

  AUCs <- lapply(p, function(p) confIntAUC(cases = p[y == 1], controls = p[y == 0]))
  result <- data.frame(CI_lower = sapply(AUCs, function(x) x$lower[2]),
                       AUC = sapply(AUCs, function(x) x$AUC[2]),
                       CI_upper = sapply(AUCs, function(x) x$upper[2]),
                       Method = names(AUCs),
                       Project = par["project"],
                       alpha = as.double(par["alpha"]))
  return(result)
}) %>%
  bind_rows() %>%
```

```r
  # same order as in tables
  mutate(Method = factor(Method, levels = levels_methods),
         Project = factor(Project, levels = levels_projects))

ggplot(auc_alpha_df, aes(x = alpha, y = AUC, color = Method)) +
  geom_hline(yintercept = 0.5, lty = 2, alpha = 0.8) +
  geom_ribbon(aes(ymin = CI_lower, ymax = CI_upper, fill = Method), alpha = 0.1, lty = 3) +
  geom_step(size = 0.6, alpha = 0.8) +
  facet_wrap(~ Project) +
  scale_x_log10(breaks = c(0.001, 0.002, 0.005, 0.015, 0.05, 0.5)) +
  labs(x = expression(alpha)) +
  theme_bw() +
  theme(legend.position = "bottom")
```

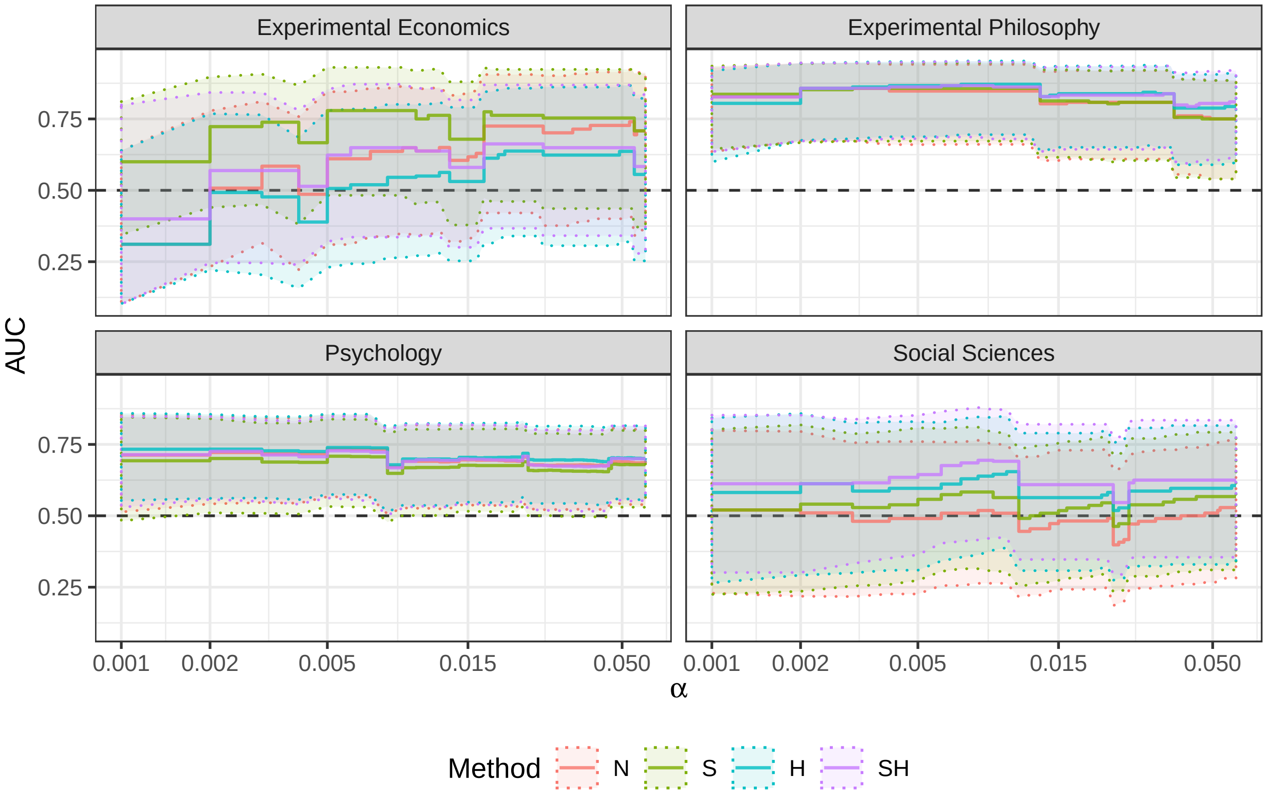

Figure 10

```r
# data from Van Erp et al. (2017) downloaded from https://osf.io/3u6dn/
tau_df <- read.csv("../../Data/tau_data_psychology.csv")
tau_cor_df <- tau_df %>%
  filter(Type.of.ES %in% c("Weighted Pearson's r",
                           "Corrected Pearson's r",
                           "Pearson's r",
                           "Weighted corrected Pearson's r"))
ggplot(tau_cor_df, aes(x = tau)) +
  geom_histogram(breaks = seq(0, 0.6, length.out = 13), col = 1, fill = "darkgrey", alpha = 0.8) +
  geom_vline(xintercept = 0.08, lty = 2, color = "darkred", size = 1, alpha = 0.8) +
  annotate(geom = "text", x = 0.16, y = 140, label = "chosen value",
           color = "darkred", size = 6) +
  geom_rug(alpha = 0.5) +
  labs(x = expression(hat(tau)), y = "Number of estimates") +
  scale_y_continuous(breaks = seq(0, 140, 20), limits = c(0, 140)) +
  theme_bw()
```

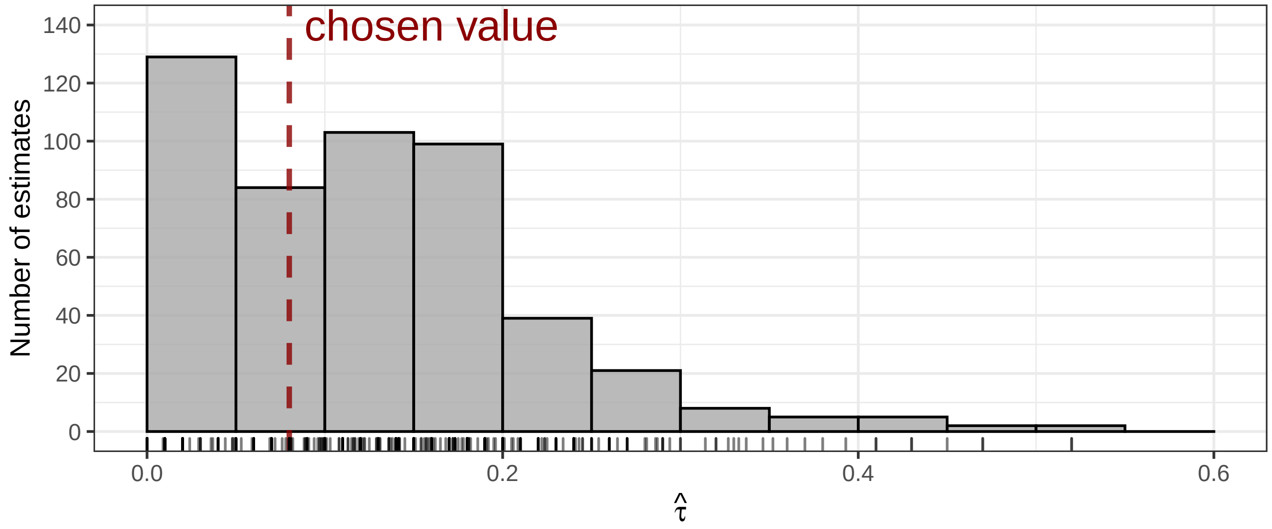

Converting pdf images to compressed tiff

```
# uses ImageMagick unix-shell program for better tiff compression than ggsave
# (otherwise files are larger than journals accept)
system(command =
       "for i in figure/*.pdf;
       do
         filename=${i%%.*};
         convert -density 320 -compress JPEG ${i} ${filename::-2}.tiff;
       done")
```

```r
sessionInfo()

## R version 3.6.2 (2019-12-12)
## Platform: x86_64-pc-linux-gnu (64-bit)
## Running under: Ubuntu 18.04.4 LTS
##
## Matrix products: default
## BLAS:   /usr/lib/x86_64-linux-gnu/openblas/libblas.so.3
## LAPACK: /usr/lib/x86_64-linux-gnu/libopenblasp-r0.2.20.so
##
## locale:
##  [1] LC_CTYPE=en_US.UTF-8       LC_NUMERIC=C
##  [3] LC_TIME=de_CH.UTF-8        LC_COLLATE=en_US.UTF-8
##  [5] LC_MONETARY=de_CH.UTF-8    LC_MESSAGES=en_US.UTF-8
##  [7] LC_PAPER=de_CH.UTF-8       LC_NAME=C
##  [9] LC_ADDRESS=C               LC_TELEPHONE=C
## [11] LC_MEASUREMENT=de_CH.UTF-8 LC_IDENTIFICATION=C
##
## attached base packages:
## [1] stats     graphics  grDevices utils     datasets  methods   base
##
## other attached packages:
##  [1] biostatUZH_1.8.0 tables_0.8.8     Hmisc_4.3-1      Formula_1.2-3
##  [5] survival_3.1-8   lattice_0.20-40  ggpubr_0.2.3     magrittr_1.5
##  [9] ggbeeswarm_0.6.0 ggplot2_3.2.1    tidyr_1.0.0      dplyr_0.8.3
## [13] knitr_1.25
##
## loaded via a namespace (and not attached):
##  [1] Rcpp_1.0.3          assertthat_0.2.1    zeallot_0.1.0
##  [4] digest_0.6.21       R6_2.4.1            plyr_1.8.5
##  [7] backports_1.1.5     acepack_1.4.1       evaluate_0.14
## [10] highr_0.8           pillar_1.4.2        rlang_0.4.2
## [13] lazyeval_0.2.2      rstudioapi_0.10     data.table_1.12.8
## [16] rpart_4.1-15        Matrix_1.2-18       checkmate_1.9.4
## [19] labeling_0.3        splines_3.6.2       stringr_1.4.0
## [22] foreign_0.8-75      htmlwidgets_1.5.1   munsell_0.5.0
## [25] compiler_3.6.2      vipor_0.4.5         xfun_0.10
## [28] pkgconfig_2.0.3     base64enc_0.1-3     htmltools_0.4.0
## [31] nnet_7.3-12         tidyselect_0.2.5    tibble_2.1.3
## [34] gridExtra_2.3       htmlTable_1.13.2    viridisLite_0.3.0
## [37] crayon_1.3.4        withr_2.1.2         grid_3.6.2
## [40] gtable_0.3.0        lifecycle_0.1.0     scales_1.0.0
## [43] stringi_1.4.3       ggsignif_0.6.0      reshape2_1.4.3
## [46] latticeExtra_0.6-28 ellipsis_0.3.0      vctrs_0.2.0
## [49] boot_1.3-24         cowplot_1.0.0       RColorBrewer_1.1-2
## [52] tools_3.6.2         glue_1.3.1          beeswarm_0.2.3
## [55] purrr_0.3.3         colorspace_1.4-1    cluster_2.1.0
```
