# Supplementary material for: Probabilistic forecasting of replication studies
Source: PLoS One. 2020 Apr 22;15(4):e0231416. doi: 10.1371/journal.pone.0231416 (PMC7176099; doi:10.1371/journal.pone.0231416)
Supplement: Attachment — Submitted filename: review_pfrs.pdf [file pone.0231416.s004.pdf]

Review of ‘Probabilistic forecasting of replication studies’, submitted to *PLOS ONE*

December 12, 2019

Summary of the paper

The replication of scientific results has recently attracted much interest by academics and the broader public. The present paper is concerned with forecasting the estimated parameter $\hat{\theta}_r$ from a replication study, based on the effect $\hat{\theta}_o$ of an associated original study. For that purpose, the paper proposes a new modeling framework that generalizes earlier work along two dimensions: First, it allows for heterogeneity between the original and replication study (arising, e.g., from a slightly different population of subjects). Second, skeptical beliefs about the underlying true parameter θ can be accommodated via an appropriate prior distribution. In an empirical analysis of four prominent replication projects, the proposed model performs well compared to a simple benchmark from the literature. The paper is well-written, and the proposed model and its evaluation are convincing.

Comments

1. While the paper's agenda is intuitively appealing, it would be worthwhile to provide a more specific motivation. Are probabilistic forecasts of replication studies interesting in their own right (and if yes, for which decision problems)? Or does the paper aim to shed light on the process that drives replicability (or lack thereof)?

2. Interestingly, the paper's prediction method uses no training data on previous pairs of replications and original studies. Instead, the link between both studies is based on the theoretical model in Equations (1a) to (1c), along with the sample sizes of the original and replication studies. This setup is quite different from most statistical forecasting applications, where the link between the outcome Y and the regressors X is typically estimated from a training sample of past data $(Y_i, X_i), i = 1, \ldots, n$. This conceptual point

is mentioned in the paper's discussion (on P20), but could be emphasized more clearly, perhaps already in the introduction.

3. On P10, the paper describes how to choose the heterogeneity variance parameter τ^2. While I understand the need for a pragmatic choice, the motivation for selecting $\tau = 0.08$ is not entirely convincing as it depends on judgmental assessments of effect sizes and a preset value for θ. Fundamentally, it seems difficult to choose τ without training data that could provide information on 'typical' differences between original and replication studies (see previous point). Is it possible to perform cross-validation in order to select τ? If yes, how does cross-validation compare to the grid of values for τ considered in Figure 10?

4. Making the four replication data sets available within an R package (as noted in the paper's conclusion) is very useful. It would also be interesting to hear some brief comments on the availability of related data sets.

Minor comments

1. P4, L76: Typo ('significt')

2. P5, L91: Typo ('analyses')

3. P5, L106: The term 'perfect separation' could be briefly explained. In particular, and unsurprisingly, the prediction market beliefs differ from the (ex post) perfect forecasts which would quote 0% for non-significant results and 100% for significant results.

4. P6, after Equations 1(a) to 1(c): It would be useful to remind the reader that σ_k^2 is simply a function of sample size in the current setup (see bottom of P3).

5. Figure 7: The legend symbol for the red outcomes (outside prediction interval) is a bit counterintuitive as it shows a dot lying within the vertical bar.

6. P24, L524: I suggest to replace 'e.g., selection bias' by a reference to the discussion in the section 'Differences between replication projects'

7. P24, L534: Perhaps replace "harder' scientific fields' by 'the life sciences'?
