# Supplementary material for: Probabilistic forecasting of replication studies
Source: PLoS One. 2020 Apr 22;15(4):e0231416. doi: 10.1371/journal.pone.0231416 (PMC7176099; doi:10.1371/journal.pone.0231416)
Supplement: Attachment — Submitted filename: ResponseToReviewers.pdf [file pone.0231416.s005.pdf]

Response to reviewers of "Probabilistic forecasting of replication studies"

Samuel Pawel, Leonhard Held
Epidemiology, Biostatistics and Prevention Institute (EBPI)
Center for Reproducible Science (CRS)
University of Zurich, Switzerland
E-mail: samuel.pawel@uzh.ch

February 25, 2020

We appreciate the comments by the reviewers. We have tried to address the comments as much as possible and have also made some additional changes and additions to the manuscript in order to improve clarity. We uploaded a version of the manuscript in which all revisions are marked, as well as an unmarked version.

Academic editor

1. *Please ensure that your manuscript meets PLOS ONE's style requirements, including those for file naming.*

   Thanks, we changed the file name of the mansucript and we also moved the table legends to below the tables. In case there is anything else that we missed, please let us know.

2. *Please provide an amended statement that declares all the funding or sources of support (whether external or internal to your organization) received during this study, as detailed online in our guide for authors at* `http://journals.plos.org/plosone/s/submit-now`*. Please also include the statement "There was no additional external funding received for this study." in your updated funding statement.*

   Thank you, we provide now an updated funding statement in the cover letter where all funding sources are declared.

Reviewer 1

The replication of scientific results has recently attracted much interest by academics and the broader public. The present paper is concerned with forecasting the estimated parameter $\hat{\theta}_r$ from a replication study, based on the effect $\hat{\theta}_o$ of an associated original study. For that purpose, the paper proposes a new modeling framework that generalizes earlier work along two dimensions: First, it allows for heterogeneity between the original and replication study (arising, e.g., from a slightly different population of subjects). Second, skeptical beliefs about the underlying true parameter θ can be accommodated via an appropriate prior distribution. In an empirical analysis of four prominent replication projects, the proposed model performs well compared to a simple benchmark from the literature. The paper is well-written, and the proposed model and its evaluation are convincing.

1. *While the paper's agenda is intuitively appealing, it would be worthwhile to provide a more specific motivation. Are probabilistic forecasts of replication studies interesting in their own right (and if yes, for which decision problems)? Or does the paper aim to shed light on the process that drives replicability (or lack thereof)?*

Thank you. We are more interested in the former for several reasons. We discuss this now more in detail in the introduction of the paper.

2. *Interestingly, the paper's prediction method uses no training data on previous pairs of replications and original studies. Instead, the link between both studies is based on the theoretical model in Equations (1a) to (1c), along with the sample sizes of the original and replication studies. This setup is quite different from most statistical forecasting applications, where the link between the outcome Y and the regressors X is typically estimated from a training sample of past data (Y_i, X_i), $i = 1, \dots, n$. This conceptual point is mentioned in the paper's discussion (on P20), but could be emphasized more clearly, perhaps already in the introduction.*

   Thanks, we added a paragraph in the introduction where this is emphasized.

3. *On P10, the paper describes how to choose the heterogeneity variance parameter τ^2. While I understand the need for a pragmatic choice, the motivation for selecting $\tau^2 = 0.08$ is not entirely convincing as it depends on judgmental assessments of effect sizes and a preset value for θ. Fundamentally, it seems difficult to choose τ without training data that could provide information on 'typical' differences between original and replication studies (see previous point). Is it possible to perform cross-validation in order to select τ? If yes, how does cross-validation compare to the grid of values for τ considered in Figure 10?*

   We appreciate this comment. As no replication estimates were used to estimate any parameter, Figure 10 provides "out-of-sample" performance measures for the grid of τ values. Hence, no cross-validation is required to find the optimal value for the data at hand. Data-driven optimum-score estimates are given by the minima of the curves. We now highlight already in the introduction that with our approach there is no need for cross-validation (in the same paragraph related to the previous comment).

4. *Making the four replication data sets available within an R package (as noted in the paper's conclusion) is very useful. It would also be interesting to hear some brief comments on the availability of related data sets.*

   The data sets from the "Many Labs" projects are also available online, *i. e.* Many Labs 1 (`https://osf.io/wx7ck/`), Many Labs 2 (`https://osf.io/8cd4r/`), and Many Labs 3 (`https://osf.io/ct89g/`). We initially considered using also these data, however, preprocessing would be much more involved and those studies are not "one-to-one", but "many-to-one" replications and therefore quite a few things in the analysis would have to be changed. This is beyond the scope of this paper, we will consider analysing Many Labs data in future work.

5. *Minor comments*

   (a) *P4, L76: Typo ('significt')*
   Corrected

   (b) *P5, L91: Typo ('analyses')*
   Corrected

   (c) *P5, L106: The term 'perfect separation' could be briefly explained. In particular, and unsurprisingly, the prediction market beliefs differ from the (ex post) perfect forecasts which would quote 0% for non-significant results and 100% for significant results.*
   We changed the name to "complete separation" and added more detailed explanations.

   (d) *P6, after Equations 1(a) to 1(c): It would be useful to remind the reader that σ_k^2 is simply a function of sample size in the current setup (see bottom of P3).*
   Added

(e) *Figure 7: The legend symbol for the red outcomes (outside prediction interval) is a bit counterintuitive as it shows a dot lying within the vertical bar.*
Corrected

(f) *P24, L524: I suggest to replace 'e.g., selection bias' by a reference to the discussion in the section 'Differences between replication projects'*
Replaced

(g) *P24, L534: Perhaps replace 'harder scientific fields' by 'the life sciences'?*
Replaced

Reviewer 2

The authors use four replication projects each containing multiple studies and compare predictive models for the effect estimates based on methods known from the probabilistic forecasting literature. The predictive models considered here allow for heterogeneity in the effect sizes between the original study and the replication and for inflated effect estimates in the original study. While the idea is interesting, I am missing a discussion of what this novel approach might add and a more detailed comparison to existing work. This leaves me wondering what the takeaway lessons might be, besides reinforcing arguments already known from the literature. I should note that my expertise lies in forecast evaluation, where no concerns arise. The technical details of the paper seem sound. I think, however, that the derivation of the "heterogeneity variance" of the effect estimates, could be explained and motivated in more detail (or changed altogether).

1. *Motivation of approach: The authors state that they "will try to predict the effect estimates of the replication studies" with a novel prediction model allowing for heterogeneity and inflation of estimates and "compare them to the forecasts from the naive model" with "established evaluation methods from the statistical prediction literature". All those tasks are well executed. I wonder, however, what the paper contributes to the broader picture. In fact, for me the most interesting result is how the prediction market fares compared to the Bayes predictions. Some statements in the abstract clearly are of general interest ("estimates from the original studies were too optimistic... some degree of heterogeneity should be expected... statistical significance as the only criterion for replication success may be questionable"). However, those have been made before and I think the article is missing an argument why the predictive comparison is an adequate tool (compared to for example a full Bayesian model) to add to those results. It strikes me as odd to construct Bayes predictions, compare different models, and then to make inference about model parameters (e.g., heterogeneity) based on predictive performance instead of estimating heterogeneity in a Bayes model. If, instead of inference about heterogeneity and inflation, the goal is the construction of a well-performing forecasting model, I would consider it more promising to have data driven models and compare them out-of-sample. Further, the authors could conclude what additional insights were gained from the more sophisticated tools (scores, PIT, etc.). The additional arguments, explaining why the approach is interesting, could be accompanied by a more detailed comparison to the existing literature. I think, for example, that Bayarri and Mayoral (2002) also employ a hierarchical model that allows for heterogeneity of effect sizes. Mentioning such similarities and pointing out differences to the existing literature would certainly improve the paper.*

   We appreciate your comments. We tried to motivate further why forecasting replication outcomes is interesting and what our approach adds to the broad picture. We also added a paragraph in the introduction where prediction markets and the need to benchmark them with statistical methods is further discussed. We now point out differences to Bayarri and Mayoral (2002) in the discussion and limitations sections. They put also priors on the variance parameters, yet use a flat prior for the underlying effect θ. This leads to no shrinkage towards zero, which is one of the main differences to our approach. In the

introduction section we now also mention the recently published study from Altmejd et al. (2019) where machine learning was used to predict replication outcomes. Finally, we now discuss in the conclusions section why more sophisticated tools should be used to evaluate probabilistic forecasts of replication outcomes.

2. *The section "Specification of the heterogeneity variance" should be improved. I have several issues with this section. First, the choice seems rather ad-hoc. I would have considered it more natural to formulate a prior over the heterogeneity parameter (or use other estimation methods), instead of assuming a fixed value. Your robustness analysis alleviates most of my concerns. So, your solution seems sensible enough, however, it took me quite some time to follow. What exactly is the "elicitation of opinion approach"? I don't think the mentioned reference gives a definition in Chapter 5.7.3, but rather applies it. I may be wrong. In any case, you could reconsider the explanation. I also think that the concept is normally meant to elicit priors from experts. I would advise to define your approach, explain it in detail, find suitable references, and discuss its implications in more detail. As part of this, let me point you to some details: (1) You write "[...] since this decision is only motivated theoretically", where I think""motivated heuristically" (or similarly) is more appropriate. (2) In line 216, "This suggests* $\tau = 0.08$ *for* $\delta(\tau)$ *being of the size of a medium effect." was confusing to me, as the argument before only discourages large effects, but not small effects. After assuming said medium effect size, you compute the respective* τ*. If this is indeed the case, I think this could be stated more explicitly. (3) It is unclear to me why the definition of effect sizes by Cohen should bear any weight in finding an appropriate variance parameter for heterogeneity. You could consider discussing this point. (4)* θ *is introduced as (underlying) effect (l. 109), later called effect size (l. 131), which is now also used for* θ_k *(l. 202). It would have been easier for me, if two different names would be used or if the symbols (*θ*,* θ_k*) would be used throughout. Finally, I should note that your results in the Section "Sensitivity analysis of heterogeneity variance choice" are insightful and a convincing argument for your choice.*

   Thank you very much for your comments. We decided against performing a full Bayesian analysis for several reasons: This would not resolve the issue of specifying hyperparameters and only add more technical complexity, because numerical or stochastic approximation would be required for the computation of the predictive distributions. With our approach it is possible to obtain them in closed-form, which allows to easily study limiting cases. We instead tried to give more details about the chosen approach and also added a new paragraph where we compare the chosen value for τ to empirical estimates from ordinary meta-analyses from psychology. The intention to conduct a sensitivity analysis is now also mentioned already before the specification approach is discussed. We agree that the term "elicitation of opinion" rather suggests prior elicitation from experts, even though it is the actual title of the chapter on which we based our approach on (Spiegelhalter et al., 2004, Chapter 5.7.3, Page 168). Therefore the term was removed. We also introduced more consistent naming of $(\theta, \theta_k, \hat{\theta}_k)$ throughout the whole paper. We used the effect size classification from Cohen because it was developed to characterize effects in psychology and other social sciences, which we now also highlight in the paper.

3. *Minor comments*

   (a) *As part of reconsidering the motivation and takeaways: The following sentence in the abstract puzzled me in the first reading. In hindsight, I know what you mean, but am still thinking this should be more precise: "...many of the estimates from the original studies were to optimistic, ...some degree of heterogeneity should be expected."*

   We tried to make the sentence more precise.

   (b) *Section numbering: I found the absence of section numbering confusing and myself often wondering if I am to embark on the next section or subsection now.*

We agree that section numbering would make everything clearer. However, the LaTeX template of PLOS ONE indicates that sections should not be numbered.

(c) *Section labels "Continuous forecasts"/"Binary Forecasts". I think it may be more helpful to name the sections differently or mention more explicitly that one considers forecasts of the effect estimates and the other forecasts of the effects being significant. Maybe the confusion arose because it is actually the target variable which is continuous/binary and not the forecast. As part of this, you might reconsider terms like "binary predictive distributions" (l. 314), which are in fact probability predictions for a binary target/outcome variable with values on the unit interval.*

We changed the section labels to "Forecasts of effect estimates" and "Forecasts of statistical significance".

(d) *line 300: The KS test specifies the behavior under uniformity, the language "test for non-uniformity" should probably be reconsidered. I have similar doubts regarding the term "miscalibration tests", which actually test the hypothesis of a calibrated forecast. I think tests are best named in accordance with their hypothesis, not the alternative they potentially have power against.*

We changed the names of the tests to "tests for uniformity" and "calibration tests".

(e) *The PIT is considered a tool for assessing calibration. It is a bit odd to start with PIT-histograms, continue with scores, before testing calibration including the PIT uniformity test. Further, including the p-values of the uniformity test in the pit-histogram discussion (or plot) seems preferable to me.*

We added the p-values of the uniformity test to the plots and removed them from the harmonic mean summary. We also moved the PIT section after the scores section.

(f) *Calibration tests: If I understand the code correctly, you use regression based calibration tests. While this is mentioned before the results in line 236, it would be great to mention this again (with more detail) in the Section "Miscalibration tests" or in the Appendix S2 (which is referred to but unfortunately does not seem to contain any more details on this point).*

We added more details about the regression tests at the beginning of the section.

(g) *Personally, I would have appreciated captions with more details for the tables, eradicating the need to search the text for definitions.*

We added more detailed explanations and abbreviation legends to the tables and figures.

(h) *line 411 - 416: I think it is more consistent to state that you assume that the effect estimate was inflated(!). The forecasting model you use therefore is shrinking ("they shrunk the effect" sounds like the initial estimate was shrunk). Also, "can be achieved" seems an overstatement. Perhaps "can be modeled" is more appropriate.*

Changed

(i) *I would like to express my compliments for providing executable code and making the data sets available via an R-package.*

Thank you

References

Altmejd, A., Dreber, A., Forsell, E., Huber, J., Imai, T., Johannesson, M., Kirchler, M., Nave, G., and Camerer, C. (2019). Predicting the replicability of social science lab experiments. *PLOS ONE*, 14(12):e0225826.

Bayarri, M. J. and Mayoral, A. M. (2002). Bayesian design of "successful" replications. *The American Statistician*, 56:207 – 214.

Spiegelhalter, D. J., Abrams, R., and Myles, J. P. (2004). *Bayesian Approaches to Clinical Trials and Health-Care Evaluation.* New York: Wiley.
